# Predicting ICU Delirium in Critically Ill COVID-19 Patients Using Demographic, Clinical, and Laboratory Admission Data: A Machine Learning Approach

**DOI:** 10.3390/life15071045

**Published:** 2025-06-30

**Authors:** Ana Viegas, Cristiana P. Von Rekowski, Rúben Araújo, Miguel Viana-Baptista, Maria Paula Macedo, Luís Bento

**Affiliations:** 1 NMS—NOVA Medical School, FCM—Faculdade de Ciências Médicas, Universidade NOVA de Lisboa, Campo dos Mártires da Pátria 130, 1169-056 Lisbon, Portugal; 2 CHRC—Comprehensive Health Research Centre, Universidade NOVA de Lisboa, Campo dos Mártires da Pátria 130, 1150-082 Lisbon, Portugal; 3 ESTeSL—Escola Superior de Tecnologia da Saúde de Lisboa, Instituto Politécnico de Lisboa, Avenida D. João II, Lote 4.69.01, Parque das Nações, 1990-096 Lisbon, Portugal; 4 H&TRC—Health & Technology Research Center, ESTeSL—Escola Superior de Tecnologia da Saúde de Lisboa, Instituto Politécnico de Lisboa, Avenida D. João II, Lote 4.69.01, Parque das Nações, 1990-096 Lisbon, Portugal; 5 Neurosciences Area, Clinical Neurophysiology Unit, ULSSJ—Unidade Local de Saúde São José, Rua José António Serrano, 1150-199 Lisbon, Portugal; 6 ISEL—Instituto Superior de Engenharia de Lisboa, Instituto Politécnico de Lisboa, Rua Conselheiro Emídio Navarro 1, 1959-007 Lisbon, Portugal; 7 Neurology Department, ULSLO—Unidade Local de Saúde de Lisboa Ocidental, Rua da Junqueira 126, 1349-019 Lisbon, Portugal; 8 CCAL—Centro Clínico Académico de Lisboa, NOVA Medical School, FCM—Faculdade de Ciências Médicas, Universidade NOVA de Lisboa, Campo dos Mártires da Pátria 130, 1169-056 Lisbon, Portugal; 9 iNOVA4Health—Advancing Precision Medicine, NOVA Medical School, FCM—Faculdade de Ciências Médicas, Universidade NOVA de Lisboa, Campo dos Mártires da Pátria 130, 1169-056 Lisbon, Portugal; 10 Intensive Care Department, ULSSJ—Unidade Local de Saúde São José, Rua José António Serrano, 1150-199 Lisbon, Portugal

**Keywords:** delirium, predictive modeling, COVID-19, SARS-CoV-2 infection, ICU, machine learning

## Abstract

Delirium is a common and underrecognized complication among critically ill patients, associated with prolonged ICU stays, cognitive dysfunction, and increased mortality. Its multifactorial causes and fluctuating course hinder early prediction, limiting timely management. Predictive models based on data available at ICU admission may help to identify high-risk patients and guide early interventions. This study evaluated machine learning models used to predict delirium in critically ill patients with SARS-CoV-2 infections using a prospective cohort of 426 patients. The dataset included demographic characteristics, clinical data (e.g., comorbidities, medication, reason for ICU admission, interventions), and routine lab test results. Five models—Logistic Regression, Support Vector Machine, Decision Tree, Random Forest, and Naïve Bayes—were developed using 112 features. Feature selection relied on Information Gain, and model performance was assessed via 10-fold cross-validation. The Naïve Bayes model showed moderate predictive performance and high interpretability, achieving an AUC of 0.717, accuracy of 65.3%, sensitivity of 62.4%, specificity of 68.1%, and precision of 66.2%. Key predictors included invasive mechanical ventilation, deep sedation with benzodiazepines, SARS-CoV-2 as the reason for ICU admission, ECMO use, constipation, and male sex. These findings support the use of interpretable models for early delirium risk stratification using routinely available ICU data.

## 1. Introduction

Delirium is a serious and common complication among critically ill patients, affecting up to 80% of mechanically ventilated intensive care unit (ICU) patients and approximately 30–50% of all ICU admissions [1,2,3]. Characterized by acute disturbances in attention, cognition, and consciousness [4], ICU delirium has been consistently associated with increased morbidity [5,6], prolonged invasive mechanical ventilation (IMV) [7,8,9], extended ICU and hospital stays [5,10], and higher mortality rates [5,7]. Beyond the immediate clinical impact, delirium contributes to long-term cognitive decline [11,12,13], post-ICU syndrome [14], and increased risk of dementia [11,15], making its prevention and early identification priorities in critical care medicine. The financial burden is also substantial, with ICU delirium contributing to higher healthcare costs due to prolonged hospitalization and increased post-discharge rehabilitation needs [5,6,16].

Despite its clinical significance, ICU delirium remains difficult to predict and diagnose [17,18,19] due to its fluctuating nature and complex etiology [20,21,22], as well as its multiple contributing factors and reliance on subjective assessment methods [23]. The lack of a universally accepted gold standard for diagnosing or predicting delirium, combined with the inherent limitations of available screening methods, further complicates accurate risk assessment [24].

Currently, the Confusion Assessment Method for the ICU (CAM-ICU) and the Intensive Care Delirium Screening Checklist (ICDSC) are widely used for bedside delirium detection [25]. These tools have shown reasonable sensitivity and specificity; however, their effectiveness relies heavily on nursing staff training, consistent patient evaluations, and interobserver reliability [26,27]. This dependence makes the assessment process time-consuming [28] and increases the risk of underdiagnosis, particularly in cases of hypoactive delirium [29]. Furthermore, most delirium screening tools, including CAM-ICU and ICDSC, focus on detection rather than prediction, limiting their role in early risk stratification and prevention [30]. Although detection tools aid in identifying delirium once it has developed, they do not proactively assess a patient’s risk before onset, thereby reducing opportunities for timely intervention.

Over the years, various predictive models have been developed in response to these challenges, evolving from traditional statistical approaches to more sophisticated machine learning (ML) techniques, to better manage and potentially mitigate the impact of delirium in ICU settings [31,32]. Historically, the prediction and management of delirium have relied on an understanding of the demographic and clinical risk factors. Traditional studies, including Logistic Regression analysis, have consistently identified several key predictors, including older age [33], male gender [8,34,35], the presence of pre-existing comorbidities [36], especially a history of dementia [37], and higher illness severity, often measured by scores such as Acute Physiology and Chronic Health Evaluation II (APACHE II) and Sequential Organ Failure Assessment (SOFA) [37,38], and the use of specific pharmacological agents, such as sedatives [8,12]. Moreover, the use of invasive medical devices and procedures such as IMV have been directly linked to an increased risk of developing delirium, highlighting the interaction between treatment modalities and patient outcomes [1,39,40].

Among the traditional predictive models, the PRE-DELIRIC [41] and E-PRE-DELIRIC [42] have been widely used. The PRE-DELIRIC model [41], a well-established ICU delirium prediction model, uses 10 clinical and laboratory parameters from the first 24 h of ICU admission and achieved AUCs of 0.87 in the development cohort and 0.84 in external validation. Similarly, the E-PRE-DELIRIC model [42] relies solely on admission data, achieving AUCs of 0.76 in the development cohort and 0.75 in the validation cohort. Both models were developed using multivariate Logistic Regression rather than advanced ML techniques; they exemplify structured, admission-based approaches that remain practical and accessible in ICU settings. Notably, a systematic review conducted by Ruppert et al. [32], which evaluated 26 ICU delirium prediction models developed over five years, found that most relied on traditional statistical techniques, particularly Logistic Regression, using data collected at a single time point, typically at ICU admission. These types of models are considered static ML approaches, as they utilize only baseline variables—without incorporating evolving clinical information throughout the ICU stay [32,42,43]. In contrast, dynamic models integrate time-varying or continuously monitored data, including physiological signals or serial laboratory results, providing the potential for the real-time updating of delirium risk. Nonetheless, their implementation in routine clinical settings can be challenging, as they often demand complex infrastructure, real-time data acquisition systems, and advanced computational resources [30,44,45,46]. Although such static models have limitations in capturing the dynamic and evolving nature of delirium risk, they continue to provide valuable clinical insights and offer a solid foundation for future approaches that incorporate more dynamic, data-driven elements. Ruppert et al. [32] also identified a growing interest in ML approaches for delirium prediction, although only a minority of models employed these techniques.

In the context of ICU delirium, ML models have the potential to integrate a wide array of inputs, including continuous physiological signals, demographic information, and structured electronic health record (EHR) data, to improve prediction accuracy [44,47]. More advanced artificial intelligence (AI) approaches further expand these capabilities by incorporating multimodal data sources, such as time series data, neuroimaging features, and the natural language processing (NLP) of clinical notes. Deep learning techniques have also been applied to automate delirium detection or prediction using clinical text, EEG, and imaging data, enabling more dynamic and comprehensive risk assessments [43]. Furthermore, recent studies have demonstrated the effectiveness of algorithms such as Random Forests, Support Vector Machines (SVMs), and deep learning architectures in enhancing delirium prediction performance across ICU and hospital settings, reinforcing the potential of ML-based approaches in clinical practice [31,43]. However, while these models leverage dynamic physiological data and deep learning techniques to improve accuracy [30,48,49], they often require continuous monitoring and greater computational resources, and they present implementation challenges in real-world ICU environments [30,46,48]. Additionally, limited generalizability and a lack of interpretability remain key barriers to their clinical adoption [44,45,46].

Given these limitations, there is growing interest in simpler, interpretable models that rely on static variables collected at ICU admission to enable early risk stratification [32,42,43]. Such models offer advantages in terms of feasibility, transparency, and integration into EHR, making them well-suited for routine clinical use [45,46,50]. Building on this approach, the present study evaluates the predictive performance of several ML algorithms using only demographic, clinical, and laboratory data available at ICU admission, with the aim of identifying effective and explainable solutions for early delirium risk detection.

ML approaches have not only revealed novel predictors but also reinforced previously established predictors of delirium, potentially enhancing risk stratification beyond traditional models. Notable physiological predictors emerging or reaffirmed by these studies include variability in heart rate [48,49], fluctuations in blood pressure, respiratory rate, and oxygen saturation [45,51]. Additionally, dynamic laboratory parameters such as changes in hemoglobin levels [45,52], electrolyte imbalances (particularly sodium and potassium) [45], renal function indicators (e.g., changes in creatinine or high blood urea nitrogen to creatinine ratio levels) [45,46,53], and inflammatory biomarkers (such as white blood cell counts and C-reactive protein) [45,54] have been highlighted as valuable for delirium prediction. Furthermore, pharmacological predictors, including the use and dosage of sedatives (e.g., benzodiazepines, propofol), opioids, and antipsychotic medications, have been consistently reinforced as influential factors in delirium development [9,55,56,57].

Despite recent advancements, delirium prediction models still face several challenges. As previously discussed, the reliance on continuously monitored data and advanced ML techniques introduces barriers related to workload [30,58], substantial computational requirements [43,59], and limited EHR integration [60]. Another critical limitation of the existing delirium prediction models is the lack of generalizability [61,62], as many models were developed and validated in specific patient populations, such as older medical [63] or surgical cohorts [64], potentially reducing their applicability to diverse ICU settings [31]. A further concern is the inherent trade-off between building complex models and ensuring that they remain interpretable. Although more advanced ML models may improve predictive performance, their “black box” nature limits their clinical adoption [65,66]. In contrast, interpretable models allow for easier implementation and integration into ICU workflows, ensuring real-time applicability. These constraints highlight the need for more effective, interpretable, and clinically applicable delirium prediction tools that facilitate early risk stratification and timely intervention.

This study explored the predictive performance of five ML models—Logistic Regression, SVM, Decision Tree, Random Forest, and Naïve Bayes—for delirium prediction at ICU admission using demographic, clinical, and laboratory data. While some prior studies have focused on using continuous monitoring data to improve prediction accuracy, others have also used structured clinical and demographic variables available at ICU admission to enable early risk stratification. This study followed the latter approach, prioritizing the early identification of high-risk patients without requiring dynamic patient updates, making it more practical for real-world ICU implementation. The objectives of this study are threefold: (1) to compare the predictive accuracy of different ML models, (2) to evaluate the trade-off between predictive performance and interpretability across different ML techniques, and (3) to explore the potential applicability of ML-based delirium prediction models for future integration into EHR-based decision-support systems. By leveraging ICU admission-only data, this study aims to contribute to the development of an efficient, interpretable, and clinically feasible prediction tool that can facilitate early intervention and improve patient outcomes in critical care settings.

## 2. Materials and Methods

### 2.1. Study Design

This prospective observational study was conducted in a tertiary care ICU in Lisbon, Portugal; it aimed to develop ML models for delirium prediction using demographic, clinical, and laboratory data collected at ICU admission from critically ill patients with SARS-CoV-2 infection. Although the study was conducted prospectively, data were extracted retrospectively from patient medical records. Data were collected from ICU admissions between 10 March 2020 and 26 August 2022.

The study was approved by the institutional ethics committee, and informed consent was obtained from all patients or their legal representatives.

### 2.2. Population

**Figure 1:**
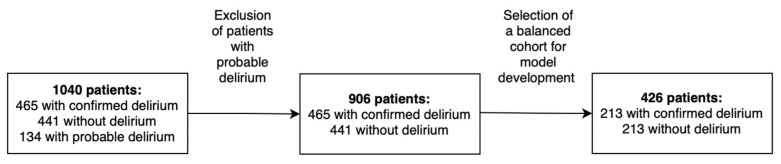
Flowchart of patient selection.

A total of 1040 adult (≥18 years) patients admitted to the ICU were included in this study (Figure 1).

Among this cohort, 465 were diagnosed with delirium, whereas 441 did not exhibit delirium, and 134 were suspected of having delirium. Probable delirium was defined as cases in which there was clinical suspicion, based on behavioral signs, medication use (e.g., neuroleptics), or medical chart notes, but where CAM-ICU assessment or formal diagnostic criteria were not consistently applied or documented. Patients classified as probable cases of delirium were excluded from the analysis to ensure that the study cohort consisted solely of cases with definitive diagnoses, minimizing potential bias and enhancing the reliability of the results. This exclusion resulted in a cohort of 906 patients. To ensure balanced group representation and minimize class imbalance bias during model development, random undersampling was applied to both groups. From the 465 patients with delirium and the 441 without, a random selection of 213 patients was generated from each class using the Balance Data widget in Orange 3 (version 3.19.0) [67], configured for undersampling without replacement. The resulting balanced dataset of 426 patients (213 with and 213 without delirium) was automatically shuffled to prevent ordering bias. This approach preserved the internal distribution of clinical features within each class while reducing bias during ML model training. Nonetheless, this strategy may introduce selection bias by excluding potentially informative data points, which could limit the generalizability of the results.

All patients tested positive for COVID-19 via real-time polymerase chain reaction targeting SARS-CoV-2. At the time of ICU admission, none of the patients were terminally ill or exhibited signs of delirium. Furthermore, the cohort was limited to patients without a history of major psychiatric disorders or pre-existing neurological conditions that might confound the analysis. To prevent potential biases from multiple admissions, only the first ICU admission for each patient during the study period was included.

Delirium was primarily assessed using the CAM-ICU method [68]. The CAM-ICU score was determined by evaluating the presence of four specific criteria to assess the patients’ cognitive state: acute onset or fluctuating course, inattention, disorganized thinking, and altered level of consciousness. Patients with a Richmond Agitation and Sedation Scale (RASS) [69] score of −4 or −5 were considered ineligible for CAM-ICU screening. Those with at least one positive CAM-ICU score during their ICU stay were diagnosed with delirium. In cases in which a delirium assessment was not performed, the delirium status was established through a comprehensive review of the clinical notes. This review assessed the use of neuroleptics, the RASS score, and the Diagnostic and Statistical Manual of the American Psychiatric Association (DSM-5) criteria [4].

### 2.3. Demographic, Clinical, and Laboratory Features

Demographic, clinical, and laboratory variables were retrospectively extracted from the electronic medical records and ICU clinical information systems. A total of 665 features were collected, corresponding to data routinely recorded at ICU admission.

Patient demographics included sex, age, and nationality. Additional contextual information included the reason for ICU admission and the specific wave of the Portuguese COVID-19 pandemic (from the first to the sixth wave) during which the patients were hospitalized.

The clinical variables encompassed a comprehensive range of comorbidities, including hypertension, constipation, diabetes mellitus, chronic kidney disease, obesity, dyslipidemia, ischemic heart disease, congestive heart failure, chronic respiratory disease, pulmonary hypertension, stroke, solid and hematologic malignancies, autoimmune disorders, chronic liver disease, history of organ transplantation, hyperuricemia, pre-existing psychiatric illness, insomnia, and neurological conditions (e.g., epilepsy, Parkinson’s disease, schizophrenia, multiple sclerosis, and amyloidosis).

Key interventions such as the use of IMV and extracorporeal membrane oxygenation (ECMO) were documented. Medication data included drugs administered both before and during the ICU stay, such as sedatives (e.g., midazolam, oxazepam), antibiotics, anticoagulants, corticosteroids, immunosuppressants, and other therapeutic classes. COVID-19-specific variables were also collected, including vaccination status and the number of days between symptom onset and ICU admission.

Laboratory parameters were obtained from routine blood tests performed at ICU admission. This included blood gas analysis, hemogram, coagulation markers (International Normalized Ratio (INR), activated partial thromboplastin time and fibrinogen), kidney function indicators (e.g., creatinine, urea), electrolytes, liver function tests (e.g., bilirubin, alkaline phosphatase, gamma-glutamyl transferase, transaminases, lactate dehydrogenase), inflammatory markers (e.g., C-reactive protein (CRP), procalcitonin and ferritin), and other biomarkers including creatine kinase and troponin.

Additional variables included the ICU length of stay and mortality rates in both the ICU and hospital settings.

All data were anonymized and compiled into a structured dataset for analysis. Data cleaning and preprocessing were performed to ensure consistency and eliminate duplicated or incomplete records. Only variables available for at least 70% of patients were considered for the ML analysis. No imputation was performed; variables slightly below this threshold were excluded to preserve data quality and avoid introducing assumptions based on incomplete information.

### 2.4. Collection of Biological Samples

Laboratory parameters were obtained from routine blood tests performed at ICU admission. For this purpose, peripheral blood was collected in a tube with no anticoagulant (VACUETTE^®^, Kremsmünster, Austria), using standard blood collection procedures. To ensure consistency, all blood collections were performed during the first morning shift in the ICU, between 7 and 9 a.m. Once collected, the blood was immediately placed in cold storage at −4 °C and processed within 2 h. The serum was then obtained by centrifugation at 3500 rpm for 10 min (Mikro 220T, Hettich, Tuttlingen, Germany) and maintained at −80 °C until further analysis.

### 2.5. Statistical Analysis

For continuous variables, comparisons between the two groups were conducted using the independent samples t-test when the data adhered to a normal distribution. In cases in which the data deviated from normality, the Mann–Whitney U test was used as a non-parametric alternative. Categorical data were analyzed using chi-square tests to assess independence within the contingency tables. When the contingency tables contained cells with low expected frequencies, Fisher’s exact test was employed to ensure accurate results. A two-sided p-value of less than 0.05 was considered indicative of statistical significance.

All descriptive and inferential analyses were performed using IBM SPSS Statistics software, version 27 (IBM Corp., New York, NY, USA).

### 2.6. ML and Data Analysis

Several ML algorithms were evaluated, including Naïve Bayes, Logistic Regression, Decision Tree, SVM, and Random Forest algorithms. The model with the highest overall performance—based on AUC, accuracy, precision, sensitivity, and specificity—was selected for detailed analysis. These metrics were chosen to provide a comprehensive evaluation of the model’s ability to distinguish between delirium and non-delirium cases. Given the potential for class imbalance, the AUC was prioritized as the primary metric because of its effectiveness in assessing the model’s discriminatory power across all decision thresholds. Additionally, sensitivity and specificity were considered to ensure a well-balanced performance and clinical applicability.

Each of the selected algorithms offers unique strengths in delirium prediction. Naïve Bayes, a probabilistic model, estimates class probabilities using Bayes’ theorem, assuming feature independence, making it computationally efficient [70,71,72]. Logistic Regression, a linear model, predicts binary outcomes using a logistic function and is valued for its simplicity and interpretability [73]. Decision Trees construct hierarchical rules for classification, allowing them to intuitively and effectively capture nonlinear relationships in data [74]. SVMs determine an optimal hyperplane that maximizes the separation margin between classes, exhibiting particular excellence with high-dimensional data when appropriate kernel functions are applied [75]. Random Forest, an ensemble learning method that aggregates multiple Decision Trees, enhances prediction stability and generalizability while mitigating overfitting by averaging outputs across the ensemble [76,77].

Feature selection was performed using the Information Gain scoring method to identify the variables most relevant for differentiating delirium from non-delirium cases. The proposed method evaluates the contribution of individual features in separating target classes [78,79]. To prevent data leakage and ensure unbiased evaluation, feature selection was integrated into the cross-validation process. The selected features were used to effectively address the research objectives. Performance metrics were obtained using 10-fold stratified cross-validation, where each fold served as a test set, while the remaining folds were used for training.

Confusion matrices were used to assess classification performance and provide insights into true positives, false positives, true negatives, and false negatives [80]. Sensitivity (recall) measured the proportion of true positive (delirium) cases correctly identified, while specificity quantified the proportion of true negative (non-delirium) cases accurately classified. These evaluations offered detailed perspectives on the data structure and clustering, which are critical for interpreting EEG features in predicting delirium.

To enhance model interpretability, a nomogram was developed for the best-performing ML model. The nomogram visually represents the influence of individual features on the predicted binary outcomes [50,81]. For binary features, it illustrates how their presence or absence affects predictions, whereas, for continuous features, predictive influences are depicted using value ranges, thereby providing insights into how different feature levels impact the outcome. By allowing users to explore the effects of feature value changes, the proposed nomogram improves the transparency and comprehension of the model’s decision-making process.

ML algorithms, feature selection, and the creation of confusion matrices and nomograms, were performed using Orange 3, version 3.19.0 (Bioinformatics Lab, University of Ljubljana, Slovenia) [67].

#### Feature Selection

To maintain the quality and relevance of the data used in the ML models, thorough criteria were applied during the preparation and refinement of both categorical and continuous variables, prior to applying the Information Gain scoring method. This process focused on removing features with low variability, inadequate representativeness, or a high percentage of missing values, as detailed below:

1. Low variability in the categorical variables:
  a. Criteria: categorical variables in which a single category (0 or 1) dominated more than 95% of the cases in both groups were excluded.
  b. Objective: to ensure that the variables exhibited sufficient variability to distinguish between the analyzed groups (delirium vs. non-delirium).
2. Low frequency and absence of categorical variables:
  a. Criteria: variables for which one category (0 or 1) had fewer than 2 absolute occurrences in either group were removed; variables for which one category was entirely absent in one group were also excluded.
  b. Objective: to ensure minimal representativeness of all categorical variables in both groups to avoid statistical bias.
3. High frequency of missing values in the continuous variables:
  a. Criteria: continuous variables with more than 30% missing values were excluded.
  b. Objective: to retain only variables with at least 70% valid values and to minimize the impact of missing data on modeling.
4. Low variance in the continuous variables:
  a. Criteria: continuous variables with a variance of less than 0.01 were removed.
  b. Objective: to eliminate features with low dispersion, which have limited predictive value for classification between groups.

These criteria were applied within the selection pipeline using Orange 3 software [67], ensuring that only the most relevant and representative features were retained for the subsequent modeling stages. This approach minimized noise, optimized data efficiency, and enhanced the predictive performance of the models while preserving the essential information for analysis. As a result, a final set of 112 demographic, clinical, and laboratory features was used to develop the ML models (Table S1).

## 3. Results

### 3.1. General Characteristics of the Population at ICU Admission

Of the 426 patients included in this study, 213 were diagnosed with delirium (Figure 1). At ICU admission, the development of delirium was significantly associated with male sex (*p* < 0.001), a longer ICU length of stay (*p* < 0.001), constipation (*p* = 0.002), use of IMV (*p* < 0.001), ECMO (*p* = 0.005), and deep sedation with (*p* < 0.001) or without benzodiazepines (*p* = 0.046). Conversely, variables such as Portuguese nationality, comorbidities (including hypertension, diabetes mellitus, dyslipidemia, and obesity), and ICU or hospital mortality were not significantly associated with delirium (all with *p* ≥ 0.05) (Table 1).

### 3.2. Demographic, Clinical, and Laboratory ML Models

To evaluate delirium risk at ICU admission, ML models were trained using demographic, clinical, and laboratory variables from patients with SARS-CoV-2 infection. Their predictive performance is presented in Table 2.

Feature selection was performed using the Information Gain scoring method, which identified the six variables with the highest predictive value for delirium. IMV emerged as the most influential predictor, followed by deep sedation with benzodiazepines, ICU admission due to SARS-CoV-2 infection, ECMO use, constipation, and male sex.

Among the evaluated models, the Naïve Bayes algorithm demonstrated the most favorable predictive performance, achieving an AUC of 0.717, which indicates moderate discrimination between delirium and non-delirium cases. This performance was further supported by consistent metrics across accuracy (65.3%), precision (66.2%), sensitivity (62.4%), and specificity (68.1%). As shown in Table 3, among the 213 patients diagnosed with delirium, the model correctly classified 66.2% (133 patients) as delirium cases (true positives), whereas 35.6% (80 patients) were misclassified as non-delirium (false negatives). For the 213 patients without delirium, 64.4% (145 patients) were correctly classified as non-delirium (true negatives), whereas 33.8% (68 patients) were incorrectly classified as delirium cases (false positives).

In practical terms, the model’s precision (66.2%) suggests that approximately two out of every three patients flagged as high risk in fact developed delirium. Its false positive rate (33.8%) is relatively modest for a screening tool and may be considered acceptable in ICU settings where the goal is to prompt timely preventive actions with minimal downsides. Collectively, these performance characteristics, although moderate, offer a useful balance between sensitivity and specificity, reinforcing the model’s potential value as an initial triage tool to identify patients who may benefit from enhanced monitoring or targeted preventive strategies. This overall profile further supports the model’s clinical applicability for early risk stratification and timely intervention in ICU settings.

Notably, despite its simplicity and assumption of feature independence [70,82], the Naïve Bayes model achieved a balanced performance comparable to, or even exceeding, that of more complex algorithms. The remaining ML models exhibited similar classification profiles, with key differences particularly in terms of the trade-off between sensitivity and specificity. For example, Logistic Regression demonstrated higher specificity (70.0%) at the cost of lower sensitivity (58.7%), reflecting a more conservative classification strategy. Conversely, the SVM model presented the opposite trend, with relatively high sensitivity (65.7%) but markedly low specificity (36.2%), indicating a limited ability to discriminate non-delirium cases. The Random Forest model, by contrast, achieved a well-balanced performance across all metrics, closely aligning with that of the Naïve Bayes algorithm.

**Figure 2:**
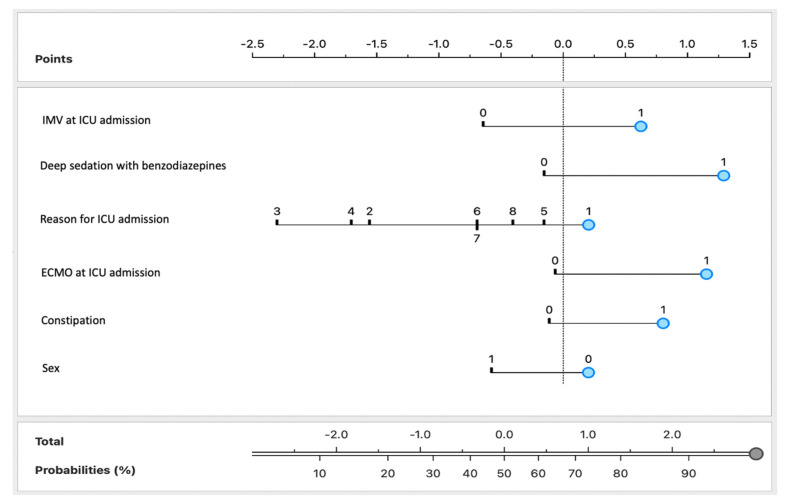
Nomogram for the Naïve Bayes model for predicting delirium based on demographic, clinical, and laboratory variables. The variables were categorized as follows: IMV use at ICU admission, deep sedation with benzodiazepines, ECMO support at ICU admission, and constipation were coded as binary variables (1 = present and 0 = absent); the reason for ICU admission was numerically coded as 1 for SARS-CoV-2 infection, 2 for urgent surgery, 3 for acute myocardial infarction, 4 for stroke, 5 for septic shock, 6 for heart rhythm changes, 7 for Guillain–Barré syndrome, and 8 for renal insufficiency; sex was coded as 0 for male and 1 for female. The blue circles indicate the binary values (0 or 1) assigned to each predictor variable.

To further enhance the clinical applicability of the Naïve Bayes model, a nomogram was developed incorporating the six most predictive features identified through Information Gain ranking. In this visual tool, each feature is assigned a corresponding score based on its presence or level, with blue markers indicating the values or categories most strongly associated with delirium development in this study cohort (Figure 2). For instance, in this study cohort, at ICU admission, a patient requiring IMV (1), deeply sedated with benzodiazepines (1), admitted due to SARS-CoV-2 infection (1), receiving ECMO support (1), presenting with constipation (1), and being male (0) would be assigned a predicted probability of approximately 95% for developing delirium. By integrating multiple risk factors into a single visual representation, this nomogram provides a user-friendly tool for individualized risk assessment, facilitating the identification of high-risk patients and allowing clinicians to implement targeted interventions and optimize ICU care strategies.

## 4. Discussion

### 4.1. Clinical and Demographic Characteristics of the Population

In this cohort, key clinical and demographic factors were associated with delirium in critically ill patients with SARS-CoV-2, while others showed no significant association, reflecting the condition’s complexity (Table 1).

Patients who developed delirium were significantly more likely to be male, to require prolonged ICU stays, and to undergo invasive interventions such as IMV and ECMO. They also had higher exposure to sedatives, particularly benzodiazepines, and a greater prevalence of gastrointestinal dysfunction, especially constipation.

These findings are consistent with prior studies demonstrating that deep sedation, particularly when involving benzodiazepines, is associated with an increased risk of delirium due to enhanced central nervous system depression and the disruption of normal cognitive processes [83,84]. This risk is further compounded by other well-established factors, including male sex [8,34,35], prolonged ICU stay [61,85], and the use of invasive interventions, such as IMV and ECMO [85,86,87], which likely reflect different but interacting pathways of vulnerability, involving illness severity, physiological stress, and sex-related biological factors. Additionally, gastrointestinal dysfunction—especially constipation—may contribute to delirium through mechanisms such as gut barrier disruption, systemic inflammation, and gut–brain axis dysregulation [88,89].

In contrast, other factors, such as Portuguese nationality, comorbidities (including hypertension, diabetes mellitus, dyslipidemia, and obesity), and ICU and hospital mortality, were not significantly associated with delirium development (all with *p* ≥ 0.05). Although prior studies have associated certain comorbidities with increased delirium risk [90,91,92,93], these reported associations were not confirmed in this study cohort’s, potentially due to differences in population characteristics or study design. Similarly, despite extensive evidence linking delirium to increased mortality [8], the chi-squared test showed no significant association between delirium and ICU mortality in this cohort, possibly due to confounding factors such as early recognition and management of delirium or differences in illness severity.

### 4.2. Performance of the Developed ML Models

This study evaluated the predictive performance of five ML algorithms—Logistic Regression, SVM, Decision Tree, Random Forest, and Naïve Bayes—for delirium prediction among critically ill patients with SARS-CoV-2 infection, using demographic, clinical, and laboratory variables collected at ICU admission.

Among the developed models, the Naïve Bayes classifier demonstrated the highest overall predictive performance, achieving an AUC of 0.717, accuracy of 65.3%, precision of 66.2%, sensitivity of 62.4%, and specificity of 68.1%. Random Forest and Decision Tree models exhibited comparable but slightly lower AUCs (0.714 and 0.707, respectively), with similar sensitivity and specificity profiles. Although these differences were modest, the simpler structure and interpretability of the Naïve Bayes algorithm [82] make it particularly attractive for clinical application, especially in environments where transparency, speed, and ease of deployment are critical.

Logistic Regression is also widely recognized for its transparency and interpretability [94], but its lower AUC (0.690) in this study suggests that the probabilistic approach of Naïve Bayes may better capture early risk patterns based on ICU admission variables. Building on this advantage, the Naïve Bayes model provides not only rapid probabilistic predictions but also a straightforward interpretation, both of which are valuable in ICU settings where timely and informed decisions can significantly affect outcomes [82]. In contrast, models such as Random Forest and Decision Tree, although generally accurate, are more complex (“black-box” models), which can limit their transparency and practical use at the bedside [94,95,96]. The SVM model, another complex approach, performed particularly poorly (AUC = 0.558), demonstrating low accuracy (50.9%) and poor specificity (36.2%), making it the least reliable model in this context. Importantly, these findings highlight that increased model complexity—characteristic of SVM and ensemble methods such as Random Forest [75,76]—does not necessarily translate into better predictive performance, especially when interpretability and clinical usability are key considerations. Although such models can capture complex, nonlinear feature interactions, their black-box nature may hinder clinical adoption, where clear, explainable reasoning remains a core requirement [94,97,98].

Balancing sensitivity and specificity remains a central challenge in the development of delirium prediction models [32,99,100]. A higher sensitivity ensures that high-risk patients are identified early and receive early intervention, whereas a lower specificity can lead to excessive false positives and unnecessary interventions. In this study, the Naïve Bayes model provided a reasonable trade-off between detecting high-risk patients (62.4% sensitivity) and minimizing false alarms (68.1% specificity). In contrast, models developed by Hur et al. [44] and Bhattacharyya et al. [30] prioritized sensitivity (>90%), but at the expense of specificity and precision, leading to a higher number of false positives and an increased clinical burden. Although these approaches effectively captured more delirium cases, the moderate specificity achieved in the present study may be more applicable in real-world ICU settings. Other studies have attempted to optimize both sensitivity and specificity. Ko et al. [101], for example, developed a delirium prediction model for cardiac ICU patients, achieving an AUC of 0.861, with a sensitivity of 83% and a specificity of 71%, thereby outperforming the Naïve Bayes model presented in this study. This superior performance may be explained by the inclusion of hemodynamic and biochemical parameters, such as albumin levels, INR, blood urea nitrogen, and C-reactive protein, which provided additional physiological context beyond the static admission data. Although such variables were included in this study’s dataset, they ranked low in the Information Gain analysis, suggesting their minimal contribution to delirium risk prediction at ICU admission and leading to their exclusion from the final models. The limited relevance of these parameters at admission underscores a potential limitation of static data and suggests that expanding the set of predictive variables to include dynamic indicators of early clinical deterioration could enhance model discrimination. However, integrating real-time physiological data poses practical challenges, including data availability issues, increased computational requirements, and potential biases introduced during feature selection [32,47,102].

Collectively, these findings emphasize the importance of balancing sensitivity and specificity while maintaining clinical interpretability. Despite its limitations, the Naïve Bayes model in this study achieved a reasonable trade-off and offers a practical approach for ICU implementation. Prior research has indicated that clinicians value model transparency in decision making, often favoring simpler models with clear reasoning over highly complex, opaque algorithms [103,104,105]. By aligning with this clinical preference, interpretable approaches such as Naïve Bayes not only facilitate adoption but also provide a solid foundation for enhancing predictive performance without compromising clarity—an essential factor in ICU settings where real-time decision making is critical.

### 4.3. Comparison with ML-Based Models

In line with previous studies applying ML for ICU delirium prediction, this study evaluated multiple ML algorithms using ICU admission-only data. A notable aspect of this work is the comprehensive comparison of the models and the use of a nomogram to enhance interpretability, thereby aiding clinical decision making. Additionally, a structured feature selection process was applied to refine the dataset to ensure that the most relevant variables contributed to the model performance.

Although this study focused on ICU admission data for early risk stratification, the fluctuating and episodic nature of delirium [8,20] makes it challenging for static models to fully capture its evolving risk. As a result, dynamic models, which incorporate continuously updated physiological data, often outperform static approaches in terms of predictive accuracy. For instance, Gong et al. [61] incorporated physiological variables updated every 12 h, achieving AUROCs between 0.785 and 0.845, and Liu et al. [106] applied a long short-term memory (LSTM)-based deep learning model, integrating sequential EHR data, yielding an AUROC of 0.952 when combined with a LightGBM model. Similarly, Bhattacharyya et al. [30] employed a rolling observation window of 12–48 h for delirium prediction, achieving an AUROC between 0.81 and 0.88, depending on the observation and prediction windows. These findings indicate that incorporating evolving clinical data can better capture the nonlinear and time-dependent patterns of delirium onset. However, as previously discussed, such methods often require continuous monitoring, extensive data infrastructure, and significant computational resources, which may not be feasible in all ICU settings.

In addition, despite the advantages of dynamic modeling, static models remain widely used in ICU settings due to their simplicity, efficiency, and interpretability, with several studies demonstrating that data collected within the first 24 h of ICU admission can yield strong predictive performance. For example, Tang et al. [107] developed an interpretable ML model incorporating demographic, disease-related, and environmental factors recorded within the first 24 h of ICU admission, achieving an AUC of 0.836, accuracy of 0.765, and sensitivity of 0.713. The DeLLiriuM model [60], trained on structured EHR data from the same period, reported AUROCs between 0.77 and 0.84. Similarly, Hur et al. [44] developed the PRIDE algorithm, utilizing Random Forest, Extreme Gradient Boosting (XGBoost), Deep Neural Networks, and Logistic Regression to predict delirium within 24 h of ICU admission, achieving an AUC of 0.82 and sensitivity of 0.76. Meanwhile, Esumi et al. [45] developed a burn patient delirium prediction model, using Logistic Regression trained solely on ICU admission variables; they reported an AUC of 0.906, outperforming other admission-based models. Although these studies demonstrate that static data, collected early in a patient’s ICU stay, can still be leveraged for predictive modeling, it is important to note the trade-offs associated with more complex approaches. Methodologies such as the large language models (LLMs) used in the DeLLiriuM model (AUROC = 0.77–0.84) [60], deep neural networks (AUROC = 0.881) [44], XGBoost (AUROC = 0.836–0.919) [44,107], and Random Forests (AUROC = 0.850–0.916) [44,45] often deliver higher accuracy but demand greater computational resources and are more challenging to interpret, which can hinder their routine clinical integration.

In contrast, although the present study’s Naïve Bayes model achieved slightly lower metrics (AUC = 0.717, accuracy = 65.3%, sensitivity = 62.4%, and specificity = 68.1%) than those reported in the previous studies, it still provides a straightforward, interpretable, and resource-efficient solution for early delirium risk stratification. Several factors may explain these performance differences, including variations in patient populations, sample sizes, feature selection methods, and dataset compositions. In addition, the exclusion of dynamic physiological markers or additional biomarkers may have contributed to the superior performance observed in other models. However, these enhancements often come at the cost of reduced interpretability and increased implementation complexity.

Moreover, although complex and advanced ML models have demonstrated superior performance in some cases, studies have also shown that they do not always translate into better clinical outcomes due to issues related to overfitting, a lack of generalizability, and computational constraints [43]. A recent systematic review [43] emphasized that many AI-based delirium prediction models reported AUCs similar to, or even lower than, that of the present study. For instance, Nagata et al. [108] developed an Extra-Trees ensemble model that achieved an AUROC of 0.76, reflecting only moderate performance despite its algorithmic complexity, and which is comparable to the present study’s performance. In comparison, a Logistic Regression-based model proposed by Cherak et al. [109] generated AUCs ranging from 0.67–0.78, further demonstrating that even traditional ML models, when properly optimized, can deliver clinically relevant accuracy without the implementation challenges associated with more complex AI approaches. Moreover, broader reviews of AI methods have highlighted the importance of transparency and usability, particularly in critical care settings where model explainability is essential for clinical adoption [110].

These comparisons illustrate the trade-offs between predictive power, complexity, and implementation feasibility observed across ML-based delirium prediction models. They also highlight the relevance of tailoring model choice to the specific clinical context and operational constraints of each ICU environment.

### 4.4. Comparison with Traditional Regression-Based Models

Compared with well-established regression-based models such as E-PRE-DELIRIC [42] and PRE-DELIRIC [41], the Naïve Bayes model developed in this study builds upon these approaches by incorporating additional laboratory features, such as arterial blood gas values, lactate, and CRP, aiming to enhance early delirium risk stratification.

The E-PRE-DELIRIC model [42], which relied solely on clinical variables collected at ICU admission, achieved an AUC of 0.76 in the development cohort and 0.75 in the validation cohort. In contrast, the PRE-DELIRIC model [41], which incorporated both clinical and laboratory data from the first 24 h of ICU stay, demonstrated higher predictive performance with an AUC of 0.87, sensitivity of 77%, and specificity of 76% in the development cohort, and an AUC of 0.84 in external validation. This superior performance may be explained by the inclusion of evolving clinical data beyond admission—capturing changes in patient status over the first 24 h—which were not available in the present study or in E-PRE-DELIRIC. Notably, despite incorporating certain laboratory features, the best model in this study demonstrated moderate performance (AUC of 0.717), although it remains clinically informative. This reinforces the idea that factors such as illness severity (e.g., APACHE-II score), neurological status (e.g., coma at admission), and metabolic markers (e.g., metabolic acidosis)—which were included in PRE-DELIRIC but not in the present study or E-PRE-DELIRIC—may play a more significant role in predicting delirium risk, as they have been consistently associated with its development.

Building on these comparisons, further insights can be gained by examining models that incorporated broader or more refined feature sets to improve prediction. Chen et al. [111] developed a Logistic Regression model using demographic, clinical, and laboratory parameters—including patient history factors such as prior delirium and dementia—achieving an AUC of 0.71, which is comparable to that of the present study’s Naïve Bayes model. Similarly, Cherak et al. [109] used the Least Absolute Shrinkage and Selection Operator (LASSO) Logistic Regression to refine feature selection, which achieved an AUC of 0.73. By applying regularization, LASSO penalizes less informative or redundant predictors, thereby improving generalizability and reducing overfitting [112,113]. In contrast, the present study used Information Gain for feature selection—a method that ranks feature importance but does not inherently remove correlated variables [102,114]—which may have diluted the overall predictive power.

Beyond admission-based models, dynamic traditional prediction models offer a complementary approach to delirium risk assessment. For example, the DYNAMIC-ICU score [115], which integrates continuously updated variables, achieved an AUC of 0.77, representing only a moderate increase over this study’s AUC of 0.717. This suggests that, while time-dependent physiological and laboratory updates may enhance predictive accuracy, the gain is limited. Moreover, as previously noted, neither static nor dynamic models necessarily outperform each other in all clinical settings—rather, they serve different operational needs. A further limitation of dynamic models is their dependence on real-time data streams, infrastructure, and considerable computational resources—requirements that may not be practical or available in all ICU environments [32,43].

These findings emphasize how model performance is closely linked to the data types, feature selection strategies, and clinical settings in which they are applied. Understanding these relationships is key to choosing appropriate models for specific operational contexts.

### 4.5. Factors Influencing Model Performance

Several factors may explain the performance differences between the present study and prior delirium prediction models. Variations in feature selection could play a role, as some models incorporated additional variables, such as pre-existing neuropsychiatric disorders [109], early ICU interventions [101,107,109,116], or severity scores [109,115]. Although these differences do not necessarily indicate a broader feature set, they may have influenced the predictive performance. Additionally, as previously noted, models such as those reported by Chen et al. (AUC = 0.71) [111] and Cherak et al. (AUC = 0.73) [109] applied more refined feature selection techniques—stepwise Logistic Regression and LASSO Regression, respectively—which may have optimized variable selection and reduced overfitting, partially accounting for their slightly higher AUCs.

Dataset characteristics also affect the model performance. Larger sample sizes, such as those used in PRE-DELIRIC (3056 patients, AUC = 0.87 development, 0.84 external validation) [41] and Cherak et al. (8878 patients, AUC up to 0.78) [109], can improve generalizability [117]. However, smaller datasets, such as those used by Chen et al. (620 patients, AUC = 0.78) [111], Li et al. (507 patients, AUC = 0.92) [118], and the present study (426 patients, AUC = 0.717), have also achieved clinically useful results, suggesting that methodological factors beyond sample size contribute to model effectiveness.

The timeframe of data collection is a critical factor in delirium prediction models. While models such as PRE-DELIRIC [41] and those in this study incorporated data from the first 24 h of ICU admission, other models, including dynamic ones such as the PRIDE model [44] and the DYNAMIC-ICU score [115], collected data at multiple time points or continuously. These dynamic approaches leverage repeated physiological measurements and evolving clinical parameters, which may improve their predictive accuracy by capturing fluctuations in delirium risk over time. In contrast, this study’s admission-based approach focused on early risk stratification, offering a practical, resource-efficient solution for identifying at-risk patients. Although it does not track evolving clinical trends, its ability to generate early insights into delirium risk provides a solid foundation for early interventions in the ICU setting.

The choice of the ML algorithm could have also influenced this study’s model performance. While Naïve Bayes was selected for its simplicity, interpretability, and low computational demands, ensemble methods and deep learning approaches have shown advantages in handling complex relationships. For instance, Ko et al. [101] (AUC = 0.861) used XGBoost along with other ML techniques, integrating biochemical and hemodynamic parameters, whereas Contreras et al. [60] (AUROC = 0.84–0.85) applied LLMs to analyze structured EHR data converted into text, a data source not available in this study. These examples highlight how both the algorithm and data type can impact performance and should be matched to the study context.

The cross-validation methodology may further contribute to AUC discrepancies. This study applied 10-fold cross-validation, which provides a more robust and stable estimate of generalizability than single train–test splits, which can artificially inflate performance metrics [119]. Standardizing the validation methodologies across the delirium prediction models would facilitate more direct performance comparisons in future research.

Taken together, these observations emphasize that methodological choices, such as the dataset size, the feature engineering strategy, the computational budget, and the validation design, directly shape reported performance and should therefore guide how delirium prediction models are benchmarked and improved in future studies.

### 4.6. Identification of Key Predictors of Delirium

Beyond model performance, this study identified several key clinical predictors of delirium at ICU admission, namely, the use of IMV, deep sedation with benzodiazepines, SARS-CoV-2 infection as the reason for ICU admission, ECMO use, constipation, and male sex. These findings are consistent with prior research and reinforce the multifactorial nature of delirium pathogenesis among critically ill patients, as discussed in detail below.

IMV emerged as the strongest predictor of ICU delirium, aligning with extensive literature [44,61,101]. The association between IMV and delirium is likely due to multiple interacting mechanisms, including hypoxia, systemic inflammation, prolonged sedation, and immobilization [1,34,120,121]. Studies have reported that up to 80% of mechanically ventilated patients develop delirium [1,56,101,122], highlighting the cognitive impact of prolonged ventilator support. The inclusion of ventilatory parameters in ML models has further reinforced the predictive value of IMV for delirium [30,44,61,101]. Furthermore, prolonged IMV has been associated with neuroinflammation, the increased permeability of the blood–brain barrier, and neurotransmitter imbalances, all of which contribute to delirium pathogenesis [34,86]. In addition, deep sedation, which is often required for patients with IMV, has been linked to prolonged ICU stays, higher mortality, and a greater risk of persistent cognitive dysfunction after hospital discharge [1,5,9,10,13]. Given these findings, early mobilization strategies, daily sedation interruptions, and ventilator weaning protocols should be prioritized to mitigate the risk of delirium in ventilated patients [86,120,123].

Deep sedation with benzodiazepines (specifically, midazolam combined with propofol, with or without fentanyl) was another significant predictor. This observation aligns with prior evidence indicating that benzodiazepine-based sedation increases the risk of delirium among critically ill patients [1,7,83,84]. Benzodiazepines and propofol exert their effects through GABAergic mechanisms, leading to cortical depression, impaired neurotransmission, and the disruption of synaptic plasticity. Additionally, several studies have reported a dose-dependent association between benzodiazepine exposure and delirium development [83]. In response to these risks, sedation strategies that minimize benzodiazepine use, favor dexmedetomidine-based protocols, and incorporate daily sedation interruptions have been widely recommended to mitigate the occurrence of delirium. By reducing sedation depth and enhancing patient interaction, these approaches may promote lighter sedation levels, facilitate early mobilization, and help preserve cognitive function during and after ICU stays.

Delirium development was also associated with ICU admission due to SARS-CoV-2 infection. COVID-19 patients requiring intensive care often exhibit heightened inflammatory responses, hypoxemia, and multi-organ dysfunction, all of which have been implicated in delirium pathogenesis [40,55,57,121]. Supporting this association, several studies conducted during the pandemic reported delirium rates ranging from 30% to 80% among critically ill patients with COVID-19 [40,57,58], emphasizing the impact of systemic inflammation and direct viral effects on the central nervous system.

In line with IMV, ECMO—another invasive life support intervention—was additionally identified as a major determinant of delirium. Patients requiring ECMO often represent the most critically ill cohort, with severe respiratory failure, prolonged sedation, immobilization, and systemic inflammation serving as key contributors to brain dysfunction [87,124,125,126]. Recent studies have shown that delirium is highly prevalent among patients with ECMO, with rates reaching over 50% in some cohorts [87,124]. Furthermore, ECMO survivors exhibit a significantly increased risk of prolonged neurocognitive impairment and neuropsychiatric sequelae after ICU discharge compared with non-ECMO patients [125,126]. These findings underscore the vulnerability of patients with ECMO to both acute brain dysfunction and long-term cognitive deficits, emphasizing the need for targeted delirium prevention and cognitive rehabilitation strategies in this population.

Continuing the multifactorial profile of delirium risk, this study also identified constipation as an important predictor, despite often being overlooked in clinical practice. Gastrointestinal dysmotility is common among critically ill patients due to opioid use, immobility, and reduced oral intake, and it has been shown to independently predict delirium [88,89]. This association may be explained by several pathophysiological mechanisms, including altered gut–brain axis communication, systemic inflammation, and the accumulation of gut-derived neurotoxins that can impair brain function.

Finally, sex-based differences also emerged, with male sex being associated with delirium. This finding, while inconsistently reported in prior studies, is supported by evidence suggesting that males may be more vulnerable to neuroinflammatory insults and ICU-related cognitive dysfunction [9,38,127].

### 4.7. Clinical Relevance

This study reinforces the multifactorial nature of ICU delirium and identifies several clinically actionable predictors at ICU admission, including IMV, deep sedation with benzodiazepines, SARS-CoV-2 infection, ECMO use, constipation, and male sex.

The association between delirium, IMV, and benzodiazepine use is consistent with the established evidence and underscores the importance of sedation-sparing strategies, early mobilization, and ventilator weaning protocols to mitigate delirium [39,83,84]. The role of SARS-CoV-2 infection in delirium risk underscores the importance of targeted monitoring during pandemics [40,55]. Similarly, ECMO use has been associated with an increased risk of delirium, reflecting the profound illness severity, prolonged sedation, immobilization, and systemic inflammation inherent to ECMO-supported patients [87]. Emerging evidence further indicates that ECMO survivors may experience persistent cognitive impairment and neuropsychiatric sequelae after ICU discharge, emphasizing the need for long-term cognitive follow-up in this population [125,126]. Constipation, though frequently underrecognized, has also emerged as a relevant predictor of delirium. Gastrointestinal dysmotility and gut–brain axis disruption may contribute to systemic inflammation and neurocognitive dysfunction, suggesting a potential mechanistic link between bowel dysfunction and delirium [88,89]. Lastly, male sex was associated with delirium risk, which is consistent with prior studies suggesting increased vulnerability to neuroinflammatory processes among males [90,91].

From a clinical perspective, although the Naïve Bayes model demonstrated moderate predictive performance (AUC = 0.717; precision = 66.2%), its interpretability, simplicity, and reliance on routinely available admission data make it a useful tool for early risk stratification in real-world ICU environments. In contrast to more complex models, the present approach aligns with typical ICU workflows by using static, early-phase data, avoiding the need for real-time integration or high technical overhead [82,128]. Despite slightly lower AUC values when compared to dynamic or ensemble methods, such as those using rolling windows or deep learning architectures [30,43,44,60,101], the strength of the Naïve Bayes model lies in its transparency, low computational demands, and ease of implementation—qualities that are especially relevant in resource-limited settings [82,94,103,104,105,110]. While the false positive rate (33.8%) indicates that some patients may be unnecessarily flagged, the low risk of harm associated with preventive measures justifies its use as a triage tool rather than a diagnostic solution. Moreover, the model’s simplicity fosters clinician trust and facilitates bedside implementation [82,94,103,104]. While advanced algorithms may offer marginal performance gains, they often require substantial infrastructure and continuous data input and introduce transparency challenges—factors that hinder clinical adoption [43,58,110,122,123].

By supporting timely and targeted clinical decisions, the Naïve Bayes model aligns with the movement toward precision ICU care and offers a potentially useful approach to reducing the burden of delirium among critically ill patients. Importantly, while COVID-19 negatively affected routine medical activity, it also catalyzed beneficial changes in ICU workflows, such as streamlined protocols and the broader adoption of digital tools, which align with the pragmatic nature of the proposed model [129].

### 4.8. Limitations

Although this study provides clinically meaningful insights, several limitations should be acknowledged to guide future improvements. First, this study used static variables available at ICU admission only, which limits the model’s ability to capture dynamic fluctuations in delirium risk during the ICU stay. Although models using dynamic data may offer improved performance, their practical implementation remains challenging and was beyond the scope of this work. Second, the analysis was limited to a single-center cohort of critically ill patients with SARS-CoV-2 infection, which may have reduced the generalizability to broader ICU populations. External validation across diverse patient groups and ICU settings is essential to confirm the applicability of the proposed approach. Third, although a wide range of clinical features was included, certain established delirium predictors—such as illness severity scores (e.g., APACHE II), pre-existing cognitive impairment or dementia, pain scores, environmental factors (e.g., noise, light exposure), and longitudinal sedation depth—were not included due to data constraints, which may have limited the model’s performance. Fourth, although Information Gain was used for feature selection and Naïve Bayes was employed as the classification model to prioritize interpretability and low computational complexity, alternative methods such as regularized regression (e.g., LASSO) or ensemble ML models could potentially capture more complex relationships without overfitting. Despite its advantages in terms of speed, simplicity, and transparency, Naïve Bayes assumes feature independence, which may oversimplify interactions among clinical variables. Future models should aim to balance interpretability and predictive performance by exploring hybrid approaches that retain clinical usability. Fifth, variables with high missingness or low variability were excluded, which, while methodologically necessary, may have inadvertently omitted potentially informative predictors. Additionally, certain clinical or contextual variables were not included in the dataset due to unavailability or inconsistent documentation in the EHR. These include potential predictors such as prior substance use (e.g., alcohol or recreational drugs), immobility due to chronic conditions or acute complications, and psychosocial stressors related to the COVID-19 pandemic (e.g., patient isolation, bereavement). Their exclusion may have limited the model’s ability to fully capture the multifactorial nature of delirium in critically ill patients. Finally, although the best-performing model in this study achieved moderate predictive performance (AUC = 0.717), its practical strength lies in providing early risk stratification at ICU admission—a time point when preventive interventions for delirium are still feasible. Nonetheless, refinement and validation are required to broaden the applicability of the proposed model. This limitation partly reflects the inherent complexity of delirium, which involves multiple interacting risk factors that are difficult to fully capture at a single time point. Pharmacologic confounding factors, particularly sedatives, analgesics, and laxatives, add further noise to the prediction process.

Overall, these limitations highlight the need for ongoing refinement and validation to optimize early risk stratification strategies in the ICU.

### 4.9. Future Research

As demonstrated in the present study, admission-based static models can already offer clinically valuable risk stratification at a time point when preventive measures are still feasible. Nonetheless, future research should focus on enhancing model accuracy while preserving clinical interpretability and feasibility. One promising direction involves the integration of dynamic, time-dependent data—such as evolving physiological markers and laboratory parameters—to better capture the fluctuating nature and temporal progression of delirium. Sequential modelling approaches, including LSTM and transformer-based networks, may improve predictive performance in this context, particularly when combined with longitudinal data that better reflect the multifactorial nature of delirium.

Additionally, external validation in broader and more diverse ICU populations—including non-COVID cohorts and surgical or neurological patients—is essential for confirming the generalizability of the proposed model. Including missing predictors such as APACHE II scores, prior cognitive impairment, and pain management may also improve future model performance. In future iterations, incorporating variable correlation analysis may further improve model performance by reducing multicollinearity and overfitting.

Beyond predictive accuracy, efforts should focus on seamless clinical integration. Embedding delirium risk models in EHR systems, paired with explainable AI tools, could facilitate real-time decision support for ICU clinicians. Finally, further investigations into novel predictors, such as gut–brain axis biomarkers and pharmacologic exposure patterns, may uncover new targets for early intervention and prevention strategies.

## 5. Conclusions

This study developed ML models for delirium prediction among critically ill patients with SARS-CoV-2 infection using demographic, clinical, and laboratory data collected at ICU admission. Among the five models developed, the Naïve Bayes algorithm demonstrated the most favorable trade-off between predictive performance and interpretability, achieving an AUC of 0.717, and supporting its potential application for early risk stratification in real-world critical care settings.

The identification of established predictors—including IMV, deep sedation with benzodiazepines, SARS-CoV-2 infection as the reason for ICU admission, ECMO use, constipation, and male sex—reinforces the multifactorial nature of delirium, spanning respiratory, hemodynamic, neurochemical, and gastrointestinal factors, and reflects the complex systemic pathophysiology underlying ICU delirium while highlighting potential pathways for early and targeted intervention.

Although the best-performing model achieved moderate performance, its clinical applicability, ease of implementation, and reliance on routinely available data make it a promising tool for integration into EHR-based clinical decision-support systems. Nevertheless, future work should focus on external validation across diverse ICU settings and the inclusion of dynamic or biomarker-based predictors to enhance generalizability and optimize predictive accuracy.

## Figures and Tables

**Table 1:** Demographic and clinical characteristics of patients with and without delirium at ICU admission.

	Patients with Confirmed Delirium (*n* = 213)	Patients Without Delirium (*n* = 213)	*p*-Value	Statistic Test
Age (years) (median, IQR)	62.00 (21.00)	62.00 (25.00)	0.377	Mann–Whitney U test
Male sex (*n*, %)	173 (81.2%)	141 (66.2%)	<0.001	Chi-square
Portuguese nationality (*n*, %)	158 (74.2%)	159 (74.6%)	0.939	Chi-square
Administration of the COVID-19 vaccine (*n*, %)	48 (22.5%)	60 (28.2%)	0.181	Chi-square
Comorbidities (*n*, %)	103 (48.4%)	86 (40.4%)	0.097	Chi-square
Hypertension (*n*, %)	109 (51.2%)	102 (47.9%)	0.498	Chi-square
Diabetes Mellitus (*n*, %)	60 (28.2%)	55 (25.8%)	0.585	Chi-square
Dyslipidemia (*n*, %)	53 (24.9%)	50 (23.5%)	0.734	Chi-square
Obesity (*n*, %)	54 (25.4%)	45 (21.1%)	0.302	Chi-square
Hospital death (*n*, %)	50 (23.5%)	50 (23.5%)	1.000	Chi-square
ICU death (*n*, %)	32 (15.0%)	41 (19.2%)	0.247	Chi-square
Days of ICU stay (median, IQR)	16.00 (14.00)	6.00 (6.00)	<0.001	Mann–Whitney U test
Use of IMV (*n*, %)	141 (66.2%)	75 (35.2%)	<0.001	Chi-square
Use of ECMO (*n*, %)	18 (8.5%)	5 (2.3%)	0.005	Chi-square
Deep sedation with benzodiazepine (*n*, %)	39 (18.3%)	10 (4.7%)	<0.001	Chi-square
Deep sedation without benzodiazepine (*n*, %)	64 (30.0%)	46 (21.6%)	0.046	Chi-square
Constipation (*n*, %)	37 (17.4%)	16 (7.5%)	0.002	Chi-square

**Table 2:** Performance metrics from the 10-fold cross-validation of the ML models developed incorporating demographic, clinical, and laboratory variables collected at ICU admission, for delirium prediction among critically ill patients with SARS-CoV-2 infection. The variables were ranked based on their predictive contributions using the Information Gain scoring method. The reported metrics include AUC, accuracy, precision, sensitivity, and specificity.

RANK (Order)	Model	AUC	Accuracy	Precision	Sensitivity	Specificity
Use of IMV Deep sedation with benzodiazepines SARS-CoV-2 as the reason for ICU admission Use of ECMO Constipation Male sex	SVM	0.558	0.509	0.507	0.657	0.362
	Logistic Regression	0.690	0.643	0.661	0.587	0.700
	Decision Tree	0.707	0.634	0.645	0.596	0.671
	Random Forest	0.714	0.655	0.660	0.638	0.671
	Naïve Bayes	0.717	0.653	0.662	0.624	0.681

**Table 3:** Confusion matrix for the Naïve Bayes delirium prediction model using demographic, clinical, and laboratory variables.

		Predicted		
Real		Without delirium	With delirium	Σ
	Without delirium	64.4% (145)	33.8% (68)	213
	With delirium	35.6% (80)	66.2% (133)	213
	Σ	225	201	426

## Data Availability

The datasets used and/or analyzed during the current study are available from the corresponding author upon reasonable request. The data are not publicly available due to their use in an ongoing research project.

## Supplementary Materials

The following supporting information can be downloaded at: https://www.mdpi.com/article/10.3390/life15071045/s1, Table S1: List of demographic, clinical, and laboratory features included in the final dataset for this study, along with their corresponding categorizations.

## References

[1] Girard T.D., Pandharipande P.P., Ely E.W. (2008). Delirium in the intensive care unit. Crit. Care.

[2] Zhang S., Cui W., Ding S., Li X., Zhang X.W., Wu Y. (2024). A cluster-randomized controlled trial of a nurse-led artificial intelligence assisted prevention and management for delirium (AI-AntiDelirium) on delirium in intensive care unit: Study protocol. PLoS One.

[3] Al Huraizi A.R., Al-Maqbali J.S., Al Farsi R.S., Al Zeedy K., Al-Saadi T., Al-Hamadani N., Al Alawi A.M. (2023). Delirium and Its Association with Short- and Long-Term Health Outcomes in Medically Admitted Patients: A Prospective Study. J. Clin. Med..

[4] American Psychiatric Association (2013). Diagnostic and Statistical Manual of Mental Disorders.

[5] Dziegielewski C., Skead C., Canturk T., Webber C., Fernando S.M., Thompson L.H., Foster M., Ristovic V., Lawlor P.G., Chaudhuri D. (2021). Delirium and Associated Length of Stay and Costs in Critically Ill Patients. Crit. Care Res. Pract..

[6] Leslie D.L., Inouye S.K. (2011). The Importance of Delirium: Economic and Societal Costs. J. Am. Geriatr. Soc..

[7] Abdelbaky A.M., Eldelpshany M.S. (2024). Patient Outcomes and Management Strategies for Intensive Care Unit (ICU)-Associated Delirium: A Literature Review. Cureus.

[8] Page V.J., Ely E.W. (2011). Delirium in Critical Care.

[9] Ely E.W., Shintani A., Truman B., Speroff T., Gordon S.M., Harrell F.E., Inouye S.K., Bernard G.R., Dittus R.S. (2004). Delirium as a Predictor of Mortality in Mechanically Ventilated Patients in the Intensive Care Unit. JAMA.

[10] Vasilevskis E.E., Chandrasekhar R., Holtze C.H., Graves J., Speroff T., Girard T.D., Patel M.B., Hughes C.G., Cao A., Pandharipande P.P. (2018). The cost of ICU delirium and coma in the intensive care unit patient. Med. Care.

[11] Goldberg T.E., Chen C., Wang Y., Jung E., Swanson A., Ing C., Garcia P.S., Whittington R.A., Moitra V. (2020). Association of Delirium With Long-term Cognitive Decline: A Meta-analysis. JAMA Neurol..

[12] Inouye S.K., Westendorp R.G.J., Saczynski J.S. (2014). Delirium in elderly people. Lancet.

[13] Girard T.D., Jackson J.C., Pandharipande P.P., Pun B.T., Thompson J.L., Shintani A.K., Gordon S.M., Canonico A.E., Dittus R.S., Bernard G.R. (2010). Delirium as a predictor of long-term cognitive impairment in survivors of critical illness. Crit. Care Med..

[14] Devlin J.W., Needham D.M. (2021). Long-term outcomes after Delirium in the ICU: Addressing gaps in our knowledge. Am. J. Respir. Crit. Care Med..

[15] Pereira J.V.B., Aung Thein M.Z., Nitchingham A., Caplan G.A. (2021). Delirium in older adults is associated with development of new dementia: A systematic review and meta-analysis. Int. J. Geriatr. Psychiatry.

[16] Oh-Park M., Chen P., Romel-Nichols V., Hreha K., Boukrina O., Barrett A.M. (2018). Delirium screening and management in inpatient rehabilitation facilities. Am. J. Phys. Med. Rehabil..

[17] Cheng H., Huang X., Yuan S., Song S., Tang Y., Ling Y., Tan S., Wang Z., Zhou F., Lyu J. (2024). Can admission Braden skin score predict delirium in older adults in the intensive care unit? Results from a multicenter study. J. Clin. Nurs..

[18] Oosterhoff J.H.F., Karhade A.V., Oberai T., Franco-Garcia E., Doornberg J.N., Schwab J.H. (2021). Prediction of Postoperative Delirium in Geriatric Hip Fracture Patients: A Clinical Prediction Model Using Machine Learning Algorithms. Geriatr. Orthop. Surg. Rehabil..

[19] Fick D.M., Auerbach A.D., Avidan M.S., Busby-Whitehead J., Ely E.W., Jones R.N., Marcantonio E.R., Needham D.M., Pandharipande P., Robinson T.N. (2017). Network for Investigation of Delirium across the U.S.: Advancing the Field of Delirium with a New Interdisciplinary Research Network. J. Am. Geriatr. Soc..

[20] Ospina J.P., King F., Madva E., Celano C.M. (2018). Clinical Medicine and Therapeutics REVIEW Epidemiology, Mechanisms, Diagnosis, and Treatment of Delirium: A Narrative Review. Clin. Med. Ther..

[21] Alexander S.K., Needham E. (2023). Diagnosis of delirium: A practical approach. Pract. Neurol..

[22] Fong T.G., Tulebaev S.R., Inouye S.K. (2009). Delirium in elderly adults: Diagnosis, prevention and treatment. Nat. Rev. Neurol..

[23] Guenther U., Weykam J., Andorfer U., Theuerkauf N., Popp J., Ely E.W., Putensen C. (2012). Implications of Objective vs Subjective Delirium Assessment in Surgical Intensive Care Patients. Am. J. Crit. Care.

[24] Moss S.J., Lee C.H., Doig C.J., Whalen-Browne L., Stelfox H.T., Fiest K.M. (2022). Delirium diagnosis without a gold standard: Evaluating diagnostic accuracy of combined delirium assessment tools. PLoS One.

[25] Gusmao-Flores D., Figueira Salluh J.I., Chalhub R.T., Quarantini L.C. (2012). The confusion assessment method for the intensive care unit (CAM-ICU) and intensive care delirium screening checklist (ICDSC) for the diagnosis of delirium: A systematic review and meta-analysis of clinical studies. Crit. Care.

[26] Brummel N.E., Vasilevskis E.E., Han J.H., Boehm L., Pun B.T., Ely E.W. (2013). Implementing Delirium Screening in the Intensive Care Unit: Secrets to Success. Crit. Care Med..

[27] Liu Y., Li Z., Li Y., Ge N., Yue J. (2023). Detecting delirium: A systematic review of ultrabrief identification instruments for hospital patients. Front. Psychol..

[28] Lian F., Li F., Tang X., Yuan Y. (2024). Risk factors for hypoactive delirium in patients with non-traumatic ARDS: A prospective, observational study. Sci. Rep..

[29] Van Eijk M.M., Van Den Boogaard M., Van Marum R.J., Benner P., Eikelenboom P., Honing M.L., Van Der Hoven B., Horn J., Izaks G.J., Kalf A. (2012). Routine Use of the Confusion Assessment Method for the Intensive Care Unit. Am. J. Respir. Crit. Care Med..

[30] Bhattacharyya A., Sheikhalishahi S., Torbic H., Yeung W., Wang T., Birst J., Duggal A., Celi L.A., Osmani V. (2022). Delirium prediction in the ICU: Designing a screening tool for preventive interventions. JAMIA Open.

[31] Strating T., Shafiee Hanjani L., Tornvall I., Hubbard R., Scott I.A. (2023). Navigating the machine learning pipeline: A scoping review of inpatient delirium prediction models. BMJ Health Care Inform..

[32] Ruppert M.M., Lipori J., Patel S., Ingersent E., Cupka J., Ozrazgat-Baslanti T., Loftus T., Rashidi P., Bihorac A. (2020). ICU Delirium Prediction Models: A Systematic Review. Crit. Care Explor..

[33] Mattar I., Fai Chan M., Childs C., Llompart-Pou A., Stover J.F. (2013). Risk Factors for Acute Delirium in Critically Ill Adult Patients: A Systematic Review. Int. Sch. Res. Notices.

[34] Hughes C.G., Pandharipande P.P., Ely E.W. (2020). Acute Brain Dysfunction in the Critically Ill.

[35] Rahimi-Bashar F., Abolhasani G., Manouchehrian N., Jiryaee N., Vahedian-Azimi A., Sahebkar A. (2021). Incidence and Risk Factors of Delirium in the Intensive Care Unit: A Prospective Cohort. Biomed. Res. Int..

[36] Zhong X., Lin J.Y., Li L., Barrett A.M., Poeran J., Mazumdar M. (2021). Derivation and validation of a novel comorbidity-based delirium risk index to predict postoperative delirium using national administrative healthcare database. Health Serv. Res..

[37] Fong T.G., Inouye S.K. (2022). The inter-relationship between delirium and dementia: The importance of delirium prevention. Nat. Rev. Neurol..

[38] Erbay Dalli Ö., Kelebek Girgin N., Kahveci F. (2023). Incidence, characteristics and risk factors of delirium in the intensive care unit: An observational study. J. Clin. Nurs..

[39] Jin T., Jin Y., Lee S.M. (2019). Medication Use and Risk of Delirium in Mechanically Ventilated Patients. Clin. Nurs. Res..

[40] Khan S.H., Lindroth H., Perkins A.J., Jamil Y., Wang S., Roberts S., Farber M., Rahman O., Gao S., Marcantonio E.R. (2020). Delirium Incidence, Duration, and Severity in Critically Ill Patients With Coronavirus Disease 2019. Crit. Care Explor..

[41] Van Den Boogaard M., Pickkers P., Slooter A.J.C., Kuiper M.A., Spronk P.E., Van Der Voort P.H.J., Van Der Hoeven J.G., Donders R., Van Achterberg T., Schoonhoven L. (2012). Development and validation of PRE-DELIRIC (PREdiction of DELIRium in ICu patients) delirium prediction model for intensive care patients: Observational multicentre study. BMJ.

[42] Wassenaar A., van den Boogaard M., van Achterberg T., Slooter A.J.C., Kuiper M.A., Hoogendoorn M.E., Simons K.S., Maseda E., Pinto N., Jones C. (2015). Multinational development and validation of an early prediction model for delirium in ICU patients. Intensive Care Med..

[43] Lv S., Li J., He H., Zhao Q., Jiang Y. (2025). Artificial Intelligence Applications in Delirium Prediction, Diagnosis, and Management: A Systematic Review. Artif. Intell. Rev..

[44] Hur S., Ko R.E., Yoo J., Ha J., Cha W.C., Chung C.R. (2021). A machine learning-based algorithm for the prediction of intensive care unit delirium (PRIDE): Retrospective study. JMIR Med. Inform..

[45] Esumi R., Funao H., Kawamoto E., Sakamoto R., Ito-Masui A., Okuno F., Shinkai T., Hane A., Ikejiri K., Akama Y. (2025). Machine Learning–Based Prediction of Delirium and Risk Factor Identification in Intensive Care Unit Patients With Burns: Retrospective Observational Study. JMIR Form. Res..

[46] Park W.R., Kim H.R., Park J.Y., Kim H.E., Cho J., Oh J. (2022). Potential Usefulness of Blood Urea Nitrogen to Creatinine Ratio in the Prediction and Early Detection of Delirium Motor Subtype in the Intensive Care Unit. J. Clin. Med..

[47] Davoudi A., Ozrazgat-Baslanti T., Ebadi A., Bursian A.C., Bihorac A., Rashidi P. (2017). Delirium Prediction using Machine Learning Models on Preoperative Electronic Health Records Data. Proc. IEEE Int. Symp. Bioinformatics Bioeng..

[48] Oh J., Cho D., Park J., Na S.H., Kim J., Heo J., Shin C.S., Kim J.J., Park J.Y., Lee B. (2018). Prediction and early detection of delirium in the intensive care unit by using heart rate variability and machine learning. Physiol. Meas..

[49] Rollo E., Marotta J., Callea A., Brunetti V., Vollono C., Scala I., Imperatori C., Frisullo G., Broccolini A., Della Marca G. (2021). Heart rate variability and delirium in acute non-cardioembolic stroke: A prospective, cross-sectional, cohort study. Neurol. Sci..

[50] Jalali A., Alvarez-Iglesias A., Roshan D., Newell J. (2019). Visualising statistical models using dynamic nomograms. PLoS ONE.

[51] Kim H., Kim M., Kim D.Y., Seo D.G., Hong J.M., Yoon D. (2024). Prediction of delirium occurrence using machine learning in acute stroke patients in intensive care unit. Front. Neurosci..

[52] Ren Y., Zhang Y., Zhan J., Sun J., Luo J., Liao W., Cheng X. (2023). Machine learning for prediction of delirium in patients with extensive burns after surgery. CNS Neurosci. Ther..

[53] Fang Y., Tang X., Gao Y., Xie H., Shen Y., Peng M., Liu J., Zhang Y., Cui Y., Xie K. (2025). Association Between Blood Urea Nitrogen and Delirium in Critically Ill Elderly Patients Without Kidney Diseases: A Retrospective Study and Mendelian Randomization Analysis. CNS Neurosci. Ther..

[54] Seo C.L., Park J.Y., Park J., Kim H.E., Cho J., Seok J.H., Kim J.J., Shin C.S., Oh J. (2021). Neutrophil-Lymphocyte Ratio as a Potential Biomarker for Delirium in the Intensive Care Unit. Front. Psychiatry.

[55] Park H.Y., Sohn H., Hong A., Han S.W., Jang Y., Yoon E.k.K., Kim M., Park H.Y. (2025). Application of machine learning for delirium prediction and analysis of associated factors in hospitalized COVID-19 patients: A comparative study using the Korean Multidisciplinary cohort for delirium prevention (KoMCoDe). Int. J. Med. Inform..

[56] Denke C., Balzer F., Menk M., Szur S., Brosinsky G., Tafelski S., Wernecke K.D., Deja M. (2018). Long-term sequelae of acute respiratory distress syndrome caused by severe community-acquired pneumonia: Delirium-associated cognitive impairment and post-traumatic stress disorder. J. Int. Med. Res..

[57] Lee S.H., Hur H.J., Kim S.N., Ahn J.H., Ro D.H., Hong A., Park H.Y., Choe P.G., Kim B., Park H.Y. (2024). Predicting delirium and the effects of medications in hospitalized COVID-19 patients using machine learning: A retrospective study within the Korean Multidisciplinary Cohort for Delirium Prevention (KoMCoDe). Digit. Health.

[58] Lindroth H., Liu K., Szalacha L., Ashkenazy S., Bellelli G., van den Boogaard M., Caplan G., Chung C.R., Elhadi M., Gurjar M. (2024). World delirium awareness and quality survey in 2023—a worldwide point prevalence study. Age Ageing.

[59] Wong A., Young A.T., Liang A.S., Gonzales R., Douglas V.C., Hadley D. (2018). Development and Validation of an Electronic Health Record–Based Machine Learning Model to Estimate Delirium Risk in Newly Hospitalized Patients Without Known Cognitive Impairment. JAMA Netw. Open.

[60] Contreras M., Kapoor S., Zhang J., Davidson A., Ren Y., Guan Z., Ozrazgat-Baslanti T., Nerella S., Bihorac A., Rashidi P. (2024). DeLLiriuM: A large language model for delirium prediction in the ICU using structured EHR. arXiv.

[61] Gong K.D., Lu R., Bergamaschi T.S., Sanyal A., Guo J., Kim H.B., Nguyen H.T., Greenstein J.L., Winslow R.L., Stevens R.D. (2023). Predicting Intensive Care Delirium with Machine Learning: Model Development and External Validation. Anesthesiology.

[62] Li H., Zang Q., Li Q., Lin Y., Duan J., Huang J., Hu H., Zhang Y., Xia D., Zhou M. (2025). Development of a machine learning-based predictive model for postoperative delirium in elderly intensive care unit patients: Retrospective Study. J. Med. Internet Res..

[63] Dylan F., Byrne G., Mudge A.M. (2019). Delirium risk in non-surgical patients: Systematic review of predictive tools. Arch. Gerontol. Geriatr..

[64] Wong C.K., Van Munster B.C., Hatseras A., Huis In ’TVeld E., Van Leeuwen B.L., De Rooij S.E., Pleijhuis R.G. (2022). Head-to-head comparison of 14 prediction models for postoperative delirium in elderly non-ICU patients: An external validation study. BMJ Open.

[65] Mi J.X., Li A.D., Zhou L.F. (2020). Review study of interpretation methods for future interpretable machine learning. IEEE Access.

[66] Matumoto K., Nohara Y., Sakaguchi M., Takayama Y., Fukushige S., Soejima H., Nakashima N. (2023). Delirium Prediction Using Machine Learning Interpretation Method and Its Incorporation into a Clinical Workflow. Appl. Sci..

[67] Demšar J., Curk T., Erjavec A., Hočevar T., Milutinovič M., Možina M., Polajnar M., Toplak M., Staric A., Črt Gorup (2013). Orange: Data Mining Toolbox in Python Tomaž Curk Matija Polajnar Laň Zagar. J. Mach. Learn. Res..

[68] Ely E.W., Inouye S.K., Bernard G.R., Gordon S., Francis J., May L., Truman B., Speroff T., Gautam S., Margolin R. (2001). Delirium in Mechanically Ventilated Patients Validity and Reliability of the Confusion Assessment Method for the Intensive Care Unit (CAM-ICU). JAMA.

[69] Sessler C.N., Gosnell M.S., Grap M.J., Brophy G.M., O’Neal P.V., Keane K.A., Tesoro E.P., Elswick R.K. (2002). The Richmond Agitation-Sedation Scale: Validity and reliability in adult intensive care unit patients. Am. J. Respir. Crit. Care Med..

[70] Taheri S., Mammadov M. (2013). Learning the naive bayes classifier with optimization models. Int. J. Appl. Math. Comput. Sci..

[71] Krishnan S. (2021). Machine learning for biomedical signal analysis. Biomedical Signal Analysis for Connected Healthcare.

[72] Marneni D., Vemula S. (2023). Analysis of COVID-19 using machine learning techniques. Statistical Modeling in Machine Learning: Concepts and Applications.

[73] Das A. (2023). Logistic Regression. Encyclopedia of Quality of Life and Well-Being Research.

[74] Zollanvari A. (2023). Decision Trees. Machine Learning with Python.

[75] Shmilovici A. (2023). Support Vector Machines. Machine Learning for Data Science Handbook: Data Mining and Knowledge Discovery Handbook.

[76] Berk R.A. (2020). Random Forests. Statistical Learning from a Regression Perspective.

[77] Sun Z., Wang G., Li P., Wang H., Zhang M., Liang X. (2024). An improved random forest based on the classification accuracy and correlation measurement of decision trees. Expert. Syst. Appl..

[78] Salama M.A., Hassan G. (2019). A Novel Feature Selection Measure Partnership-Gain. Int. J. Online Biomed. Eng. (IJOE).

[79] Fahrudy D., Uyun S. (2022). Classification of Student Graduation using NaÃ¯ve Bayes by Comparing between Random Oversampling and Feature Selections of Information Gain and Forward Selection. JOIV Int. J. Inform. Vis..

[80] Singh P., Singh N., Singh K.K., Singh A. (2021). Diagnosing of disease using machine learning. Machine Learning and the Internet of Medical Things in Healthcare.

[81] Vellido A. (2020). The importance of interpretability and visualization in machine learning for applications in medicine and health care. Neural Comput. Applic.

[82] Wang J.W.D. (2025). Naïve Bayes is an interpretable and predictive machine learning algorithm in predicting osteoporotic hip fracture in-hospital mortality compared to other machine learning algorithms. PLoS Digit. Health.

[83] Van Gelder T.G., van Diem-Zaal I.J., Dijkstra-Kersten S.M.A., de Mul N., Lalmohamed A., Slooter A.J.C. (2024). The risk of delirium after sedation with propofol or midazolam in intensive care unit patients. Br. J. Clin. Pharmacol..

[84] Yang J., Zhou Y., Kang Y., Xu B., Wang P., Lv Y., Wang Z. (2017). Risk Factors of Delirium in Sequential Sedation Patients in Intensive Care Units. Biomed Res. Int..

[85] Viegas A., Araújo R., Ramalhete L., Von Rekowski C., Fonseca T.A.H., Bento L., Calado C.R.C. (2024). Discovery of Delirium Biomarkers through Minimally Invasive Serum Molecular Fingerprinting. Metabolites.

[86] Kotfis K., Williams Roberson S., Wilson J.E., Dabrowski W., Pun B.T., Ely E.W. (2020). COVID-19: ICU delirium management during SARS-CoV-2 pandemic. Crit. Care.

[87] Krupa S., Friganovic A., Mędrzycka-Dabrowska W. (2021). Occurrence of delirium during ecmo therapy in a critical care unit in poland—A cross-sectional pilot study. Int. J. Environ. Res. Public Health.

[88] Smonig R., Wallenhorst T., Bouju P., Letheulle J., Le Tulzo Y., Tadié J.M., Gacouin A. (2016). Constipation is independently associated with delirium in critically ill ventilated patients. Intensive Care Med..

[89] Ticinesi A., Parise A., Nouvenne A., Cerundolo N., Prati B., Meschi T. (2023). The possible role of gut microbiota dysbiosis in the pathophysiology of delirium in older persons. Microbiome Res. Rep..

[90] Ohl I.C.B., Chavaglia S.R.R., Ohl R.I.B., Lopes M.C.B.T., Campanharo C.R.V., Okuno M.F.P., Batista R.E.A. (2019). Evaluation of delirium in aged patients assisted at emergency hospital service. Rev. Bras. Enferm..

[91] Li H.-R., Guo Y. (2024). High-risk factors for delirium in severely ill patients and the application of emotional nursing combined with pain nursing. World J. Psychiatry.

[92] Kotfis K., Szylińska A., Listewnik M., Brykczyński M., Ely E.W., Rotter I. (2019). Diabetes and elevated preoperative HbA1c level as risk factors for postoperative delirium after cardiac surgery: An observational cohort study. Neuropsychiatr. Dis. Treat..

[93] Kong D., Luo W., Zhu Z., Sun S., Zhu J. (2022). Factors associated with post-operative delirium in hip fracture patients: What should we care. Eur. J. Med. Res..

[94] Lu S.C., Swisher C.L., Chung C., Jaffray D., Sidey-Gibbons C. (2023). On the importance of interpretable machine learning predictions to inform clinical decision making in oncology. Front. Oncol..

[95] Wallace M.L., Mentch L., Wheeler B.J., Tapia A.L., Richards M., Zhou S., Yi L., Redline S., Buysse D.J. (2023). Use and misuse of random forest variable importance metrics in medicine: Demonstrations through incident stroke prediction. BMC Med. Res. Methodol..

[96] Lundberg S.M., Erion G., Chen H., DeGrave A., Prutkin J.M., Nair B., Katz R., Himmelfarb J., Bansal N., Lee S.-I. (2020). Explainable AI for Trees: From Local Explanations to Global Understanding. Nat. Mach. Intell..

[97] Petkovic D., Altman R., Wong M., Vigil A. (2018). Improving the explainability of Random Forest classifier—User centered approach. Pac. Symp. Biocomput..

[98] Gao X., Alam S., Shi P., Dexter F., Kong N. (2023). Interpretable machine learning models for hospital readmission prediction: A two-step extracted regression tree approach. BMC Med. Inform. Decis. Mak..

[99] Heinrich M., Woike J.K., Spies C.D., Wegwarth O. (2022). Forecasting Postoperative Delirium in Older Adult Patients with Fast-and-Frugal Decision Trees. J. Clin. Med..

[100] Amerongen Hvan N., Stapel S., Spijkstra J.J., Ouweneel D., Schenk J. (2023). Comparison of Prognostic Accuracy of 3 Delirium Prediction Models. Am. J. Crit. Care.

[101] Ko R.-E., Lee J., Kim S., Ahn J.H., Na S.J., Yang J.H. (2024). Machine learning methods for developing a predictive model of the incidence of delirium in cardiac intensive care units. Rev. Española Cardiol. (Engl. Ed.).

[102] Pudjihartono N., Fadason T., Kempa-Liehr A.W., O’Sullivan J.M. (2022). A Review of Feature Selection Methods for Machine Learning-Based Disease Risk Prediction. Front. Bioinform..

[103] Rudin C. (2018). Stop Explaining Black Box Machine Learning Models for High Stakes Decisions and Use Interpretable Models Instead. Nat. Mach. Intell..

[104] Tonekaboni S., Joshi S., McCradden M.D., Goldenberg A. (2019). What Clinicians Want: Contextualizing Explainable Machine Learning for Clinical End Use. Proc. Mach. Learn. Res..

[105] Elshawi R., Al-Mallah M.H., Sakr S. (2019). On the interpretability of machine learning-based model for predicting hypertension. BMC Med. Inform. Decis. Mak..

[106] Liu S., Schlesinger J.J., McCoy A.B., Reese T.J., Steitz B., Russo E., Koh B., Wright A. (2023). New onset delirium prediction using machine learning and long short-Term memory (LSTM) in electronic health record. J. Am. Med. Inform. Assoc..

[107] Tang D., Ma C., Xu Y. (2024). Interpretable machine learning model for early prediction of delirium in elderly patients following intensive care unit admission: A derivation and validation study. Front. Med..

[108] Nagata C., Hata M., Miyazaki Y., Masuda H., Wada T., Kimura T., Fujii M., Sakurai Y., Matsubara Y., Yoshida K. (2023). Development of postoperative delirium prediction models in patients undergoing cardiovascular surgery using machine learning algorithms. Sci. Rep..

[109] Cherak S.J., Soo A., Brown K.N., Wesley Ely E., Stelfox H.T., Fiest K.M. (2020). Development and validation of delirium prediction model for critically ill adults parameterized to ICU admission acuity. PLoS ONE.

[110] Hassija V., Chamola V., Mahapatra A., Singal A., Goel D., Huang K., Scardapane S., Spinelli I., Mahmud M., Hussain A. (2024). Interpreting Black-Box Models: A Review on Explainable Artificial Intelligence. Cognit. Comput..

[111] Chen Y., Du H., Wei B.H., Chang X.N., Dong C.M. (2017). Development and validation of risk-stratification delirium prediction model for critically ill patients. Medicine.

[112] Alhamzawi R., Ali H.T.M. (2018). The Bayesian adaptive lasso regression. Math. Biosci..

[113] Friedman J., Hastie T., Tibshirani R. (2010). Regularization Paths for Generalized Linear Models via Coordinate Descent. J. Stat. Softw..

[114] Li C., Wang X. A Feature Selection Method Based on Competition Winners Mechanism. Proceedings of the 2015 International Power, Electronics and Materials Engineering Conference.

[115] Fan H., Ji M., Huang J., Yue P., Yang X., Wang C., Ying W. (2019). Development and validation of a dynamic delirium prediction rule in patients admitted to the Intensive Care Units (DYNAMIC-ICU): A prospective cohort study. Int. J. Nurs. Stud..

[116] Kim J.H., Hua M., Whittington R.A., Lee J., Liu C., Ta C.N., Marcantonio E.R., Goldberg T.E., Weng C. (2022). A machine learning approach to identifying delirium from electronic health records. JAMIA Open.

[117] Andrade C. (2020). Sample Size and its Importance in Research. Indian J. Psychol. Med..

[118] Li Q., Li J., Chen J., Zhao X., Zhuang J., Zhong G., Song Y., Lei L. (2024). A machine learning-based prediction model for postoperative delirium in cardiac valve surgery using electronic health records. BMC Cardiovasc. Disord..

[119] Ghasemzadeh H., Hillman R.E., Mehta D.D. (2023). Toward Generalizable Machine Learning Models in Speech, Language, and Hearing Sciences: Estimating Sample Size and Reducing Overfitting. J. Speech Lang. Hear. Res..

[120] Park S.Y., Lee H.B. (2019). Prevention and management of delirium in critically ill adult patients in the intensive care unit: A review based on the 2018 PADIS guidelines. Acute Crit. Care.

[121] Liu S.B., Wu H.Y., Duan M.L., Yang R.L., Ji C.H., Liu J.J., Zhao H. (2024). Delirium in the ICU: How much do we know? A narrative review. Ann. Med..

[122] Pun B., Devlin J. (2013). Delirium monitoring in the ICU: Strategies for initiating and sustaining screening efforts. Semin. Respir. Crit. Care Med..

[123] Eldean T.N.N., Bakri M.H., Aziz M.A.A., Khalaf G.S. (2024). Effectiveness of the ABCDEF Bundle to Manage and Prevent Delirium Pre- and Postintervention Quasi-Experimental Study. Crit. Care Nurs. Q..

[124] Ho M.H., Lee J.J., Lai P.C.K., Li P.W.C. (2023). Prevalence of delirium among critically ill patients who received extracorporeal membrane oxygenation therapy: A systematic review and proportional meta-analysis. Intensive Crit. Care Nurs..

[125] Kalra A., Kang J.K., Khanduja S., Menta A.K., Ahmad S.A., Liu O., Rodriguez E., Spann M., Hernandez A.V., Brodie D. (2024). Long-Term Neuropsychiatric, Neurocognitive and Functional Outcomes of Patients Receiving ECMO: A Systematic Review and Meta-Analysis. Neurology.

[126] Fernando S.M., Scott M., Talarico R., Fan E., McIsaac D.I., Sood M.M., Myran D.T., Herridge M.S., Needham D.M., Hodgson C.L. (2022). Association of Extracorporeal Membrane Oxygenation with New Mental Health Diagnoses in Adult Survivors of Critical Illness. JAMA.

[127] Al-Hoodar R.K., Lazarus E.R., Al Omari O., Al Zaabi O. (2022). Incidence, Associated Factors, and Outcome of Delirium among Patients Admitted to ICUs in Oman. Crit. Care Res. Pract..

[128] Cui Z., Fritz B.A., King C.R., Avidan M.S., Chen Y. (2020). A Factored Generalized Additive Model for Clinical Decision Support in the Operating Room. AMIA Annu. Symp. Proc..

[129] Siragusa L., Angelico R., Angrisani M., Zampogna B., Materazzo M., Sorge R., Giordano L., Meniconi R., Coppola A., SPIGC Survey Collaborative Group (2023). How future surgery will benefit from SARS-CoV-2-related measures: A SPIGC survey conveying the perspective of Italian surgeons. Updates Surg..

