# Supplementary material for: Predicting ICU Delirium in Critically Ill COVID-19 Patients Using Demographic, Clinical, and Laboratory Admission Data: A Machine Learning Approach
Source: Life (Basel). 2025 Jun 30;15(7):1045. doi: 10.3390/life15071045 (PMC12299776; doi:10.3390/life15071045)
Supplement: Supplementary file 1 [file life-15-01045-s001.zip › life-3704550-supplementary.pdf]

life

MDPI

Article

Predicting ICU Delirium in Critically Ill COVID-19 Patients Using Demographic, Clinical, and Laboratory Admission Data: A Machine Learning Approach

Ana Viegas [1,2,3,4,5,*], Cristiana P. Von Rekowski [1,2,6], Rúben Araújo [1,2,6], Miguel Viana-Baptista [1,7,8], Maria Paula Macedo [1,9] and Luís Bento [1,2,10,*]

[1] NMS—NOVA Medical School, FCM—Faculdade de Ciências Médicas, Universidade NOVA de Lisboa, Campo dos Mártires da Pátria 130, 1169-056 Lisbon, Portugal; crisvr66@hotmail.com (C.P.V.R.); rubenalexandredinisaraujo@gmail.com (R.A.); mvianabaptista@nms.unl.pt (M.V.-B.); paula.macedo@nms.unl.pt (M.P.M.)

[2] CHRC—Comprehensive Health Research Centre, Universidade NOVA de Lisboa, Campo dos Mártires da Pátria 130, 1150-082 Lisbon, Portugal

[3] ESTeSL—Escola Superior de Tecnologia da Saúde de Lisboa, Instituto Politécnico de Lisboa, Avenida D. João II, Lote 4.69.01, Parque das Nações, 1990-096 Lisbon, Portugal

[4] H&TRC—Health & Technology Research Center, ESTeSL—Escola Superior de Tecnologia da Saúde de Lisboa, Instituto Politécnico de Lisboa, Avenida D. João II, Lote 4.69.01, Parque das Nações, 1990-096 Lisbon, Portugal

[5] Neurosciences Area, Clinical Neurophysiology Unit, ULSSJ—Unidade Local de Saúde São José, Rua José António Serrano, 1150-199 Lisbon, Portugal

[6] ISEL—Instituto Superior de Engenharia de Lisboa, Instituto Politécnico de Lisboa, Rua Conselheiro Emídio Navarro 1, 1959-007 Lisbon, Portugal

[7] Neurology Department, ULSLO—Unidade Local de Saúde de Lisboa Ocidental, Rua da Junqueira 126, 1349-019 Lisbon, Portugal

[8] CCAL—Centro Clínico Académico de Lisboa, NOVA Medical School, FCM—Faculdade de Ciências Médicas, Universidade NOVA de Lisboa, Campo dos Mártires da Pátria 130, 1169-056 Lisbon, Portugal

[9] iNOVA4Health—Advancing Precision Medicine, NOVA Medical School, FCM—Faculdade de Ciências Médicas, Universidade NOVA de Lisboa, Campo dos Mártires da Pátria 130, 1169-056 Lisbon, Portugal

[10] Intensive Care Department, ULSSJ—Unidade Local de Saúde São José, Rua José António Serrano, 1150-199 Lisbon, Portugal

* Correspondence: a2020449@nms.unl.pt (A.V.); luis.bento@ulssjose.min-saude.pt (L.B.)

 https://doi.org/10.3390/life15071045

Supplementary Materials:

Table S1: List of demographic, clinical, and laboratory features included in the final dataset for this study, along with their corresponding categorizations.

**Feature**	**Categorization**
Age	Numerical (years)
Alanine Aminotransferase (maximum)	Numerical (U/L)
Alanine Aminotransferase (minimum)	Numerical (U/L)
Alkaline Phosphatase (maximum)	Numerical (U/L)
Alkaline Phosphatase (minimum)	Numerical (U/L)
Alprazolam (therapeutics)	1 – Present; 0 – Absent
Amlodipine (therapeutics)	1 – Present; 0 – Absent
Amoxicillin + Clavulanic Acid (therapeutics)	1 – Present; 0 – Absent
Arterial Blood Gas ǀ Lactate (maximum)	Numerical (mmol/L)
Arterial Blood Gas ǀ Lactate (minimum)	Numerical (mmol/L)
Arterial Blood Gas ǀ pCO_2 (minimum)	Numerical (mmHg)
Arterial Blood Gas ǀ pCO_2 (maximum)	Numerical (mmHg)
Arterial Blood Gas ǀ pO_2 (maximum)	Numerical (mmHg)
Arterial Blood Gas ǀ pO_2 (minimum)	Numerical (mmHg)
Hypertension	1 – Present; 0 – Absent
Aspartate Aminotransferase (maximum)	Numerical (U/L)
Aspartate Aminotransferase (minimum)	Numerical (U/L)
Atorvastatin (therapeutics)	1 – Present; 0 – Absent
Azithromycin (therapeutics)	1 – Present; 0 – Absent
Beclomethasone (therapeutics)	1 – Present; 0 – Absent
Bisoprolol (therapeutics)	1 – Present; 0 – Absent
Budesonide (therapeutics)	1 – Present; 0 – Absent
C-reactive Protein (maximum)	Numerical (mg/L)
C-reactive Protein (minimum)	Numerical (mg/L)
Calcium (maximum)	Numerical (mg/dL)
Calcium (minimum)	Numerical (mg/dL)
Calcium Gluconate (therapeutics)	1 – Present; 0 – Absent
Captopril (therapeutics)	1 – Present; 0 – Absent
Cardiac Dysrhythmia	1 – Present; 0 – Absent
Ceftriaxone (therapeutics)	1 – Present; 0 – Absent
Chronic Kidney Disease	1 – Present; 0 – Absent
Chronic Respiratory Disease	1 – Present; 0 – Absent
COVID-19 vaccination status	1 – Present; 0 – Absent
Creatine Kinase (maximum)	Numerical (U/L)
Creatine Kinase (minimum)	Numerical (U/L)
Creatinine (maximum)	Numerical (mg/dL)
Creatinine (minimum)	Numerical (mg/dL)

Days between COVID-19 symptom onset and ICU admission	Numerical (days)
Deep sedation with benzodiazepines (therapeutics)	1 – Present; 0 – Absent
Deep sedation without benzodiazepines (therapeutics)	1 – Present; 0 – Absent
Dexamethasone	1 – Present; 0 – Absent
Diabetes	1 – Present; 0 – Absent
Dyslipidemia	1 – Present; 0 – Absent
ECMO on ICU admission	1 – Present; 0 – Absent
Electrolytes (therapeutics)	1 – Present; 0 – Absent
Electrolytes + Glucose (therapeutics)	1 – Present; 0 – Absent
Enoxaparin Sodium (therapeutics)	1 – Present; 0 – Absent
Erythrocytes (maximum)	Numerical ($\times 10^{12}$/L)
Erythrocytes (minimum)	Numerical ($\times 10^{12}$/L)
Furosemide (therapeutics)	1 – Present; 0 – Absent
Gamma-Glutamyl Transferase (maximum)	Numerical (U/L)
Gamma-Glutamyl Transferase (minimum)	Numerical (U/L)
Glucose (therapeutics)	1 – Present; 0 – Absent
Hematocrit (maximum)	Numerical (%)
Hematocrit (minimum)	Numerical (%)
Hematologic Cancer	1 – Present; 0 – Absent
Hemoglobin (maximum)	Numerical (g/dL)
Hemoglobin (minimum)	Numerical (g/dL)
Hydrocortisone (therapeutics)	1 – Present; 0 – Absent
IMV on ICU admission	1 – Present; 0 – Absent
Insulin (therapeutics)	1 – Present; 0 – Absent
Ionogram ∣ Chloride (maximum)	Numerical (mEq/L)
Ionogram ∣ Chloride (minimum)	Numerical (mEq/L)
Ionogram ∣ Potassium (maximum)	Numerical (mEq/L)
Ionogram ∣ Potassium (minimum)	Numerical (mEq/L)
Ionogram ∣ Sodium (maximum)	Numerical (mEq/L)
Ionogram ∣ Sodium (minimum)	Numerical (mEq/L)
Ipratropium Bromide (therapeutics)	1 – Present; 0 – Absent
Ischemic Heart Disease	1 – Present; 0 – Absent
Ketamine (therapeutics)	1 – Present; 0 – Absent
Lactate Dehydrogenase (maximum)	Numerical (U/L)
Lactate Dehydrogenase (minimum)	Numerical (U/L)
Leukocytes (maximum)	Numerical ($\times 10^{9}$/L)
Leukocytes (minimum)	Numerical ($\times 10^{9}$/L)
Lorazepam (therapeutics)	1 – Present; 0 – Absent
Lymphocytes (maximum)	Numerical ($\times 10^{9}$/L)
Lymphocytes (minimum)	Numerical ($\times 10^{9}$/L)

Magnesium Metamizole (therapeutics)	1 – Present; 0 – Absent
Methylprednisolone (therapeutics)	1 – Present; 0 – Absent
Metoclopramide (therapeutics)	1 – Present; 0 – Absent
Monopotassium Phosphate (therapeutics)	1 – Present; 0 – Absent
Morphine (therapeutics)	1 – Present; 0 – Absent
Nationality	1 – Portugal<br>2 – African countries<br>3 – Asian countries<br>4 – Other European countries<br>5 – South American countries<br>6 – North American countries
Neutrophils (maximum)	Numerical ($\times 10^9$/L)
Neutrophils (minimum)	Numerical ($\times 10^9$/L)
Norepinephrine (therapeutics)	1 – Present; 0 – Absent
Obesity	1 – Present; 0 – Absent
Obstipation	1 – Present; 0 – Absent
Pantoprazole (therapeutics)	1 – Present; 0 – Absent
Paracetamol (therapeutics)	1 – Present; 0 – Absent
Piperacillin + Tazobactam (therapeutics)	1 – Present; 0 – Absent
Platelets (maximum)	Numerical ($\times 10^9$/L)
Platelets (minimum)	Numerical ($\times 10^9$/L)
Potassium Chloride	1 – Present; 0 – Absent
Prothrombin Time (maximum)	Numerical (INR)
Prothrombin Time (minimum)	Numerical (INR)
Quetiapine (therapeutics)	1 – Present; 0 – Absent
Reason for ICU admission	1 – Acute respiratory failure due to COVID-19<br>2 – Urgent surgery<br>3 – Acute myocardial infarction<br>4 – Stroke<br>5 – Septic shock<br>6 – Heart rhythm changes<br>7 – Guillain-Barré syndrome<br>8 – Renal insufficiency
Remdesivir (therapeutics)	1 – Present; 0 – Absent
Rocuronium Bromide (therapeutics)	1 – Present; 0 – Absent
Salbutamol (therapeutics)	1 – Present; 0 – Absent
Sex	1 – Female; 0 – Male
Sodium Chloride	1 – Present; 0 – Absent
Solid Cancer	1 – Present; 0 – Absent
Total Bilirubin (maximum)	Numerical (mg/dL)
Total Bilirubin (minimum)	Numerical (mg/dL)
Troponin I | High Sensitivity (maximum)	Numerical (pg/mL)

Troponin I | High Sensitivity (minimum)	Numerical (pg/mL)
Urea (maximum)	Numerical (mg/dL)
Urea (minimum)	Numerical (mg/dL)
Vancomycin (therapeutics)	1 – Present; 0 – Absent
